# An International Collaborative Initiative to Establish a Quality-of-Life Questionnaire for Children and Adolescents with Repair of Esophageal Atresia in 14 Countries

**DOI:** 10.3390/children11030286

**Published:** 2024-02-26

**Authors:** The International EA-QOL Group

**Affiliations:** 1 The Department of Pediatrics, Institute of Clinical Sciences, University of Gothenburg, 40530 Gothenburg, Sweden; 2 The Department of Pediatric Surgery, Queen Silvia Children’s Hospital, 41685 Gothenburg, Sweden; 3 Department of Women’s and Children’s Health, Karolinska Institutet, 17177 Stockholm, Sweden

**Keywords:** quality of life, esophageal atresia, cognitive debriefing interview, children, cultural adaptation, rare disease

## Abstract

The EA-QOL questionnaire measures quality-of-life specifically for children born with esophageal atresia (EA) aged 8–18 and was completed in Sweden and Germany. This study aimed to describe an international collaborative initiative to establish a semantically equivalent linguistic version of the EA-QOL questionnaires in 12 new countries. The 24-item EA-QOL questionnaire was translated into the target languages and the translated questionnaire was evaluated through cognitive debriefing interviews with children with EA aged 8–18 and their parents in each new country. Participants rated an item as to whether an item was easy to understand and sensitive/uncomfortable to answer. They could choose not to reply to a non-applicable/problematic item and provide open comments. Data were analyzed using predefined psychometric criteria; item clarity ≥80%, item sensitive/uncomfortable to answer ≤20%, item feasibility(missing item responses ≤5%). Decision to improve any translation was made by native experts–patient stakeholders and the instrument developer. Like in Sweden and Germany, all items in the cross-cultural analysis of child self-report (*n*_tot_ = 82, 4–10 children/country) met the criteria for item clarity in all 12 new countries, and in parent-report (*n*_tot_ = 86, 5–10 parents/country) in 8/12 countries. All items fulfilled the criteria for sensitive/uncomfortable to answer (child-report 1.2–9.9%; parent-report 0–11.6%) and item feasibility. Poor translations were resolved. Hence, this study has established semantically equivalent linguistic versions of the EA-QOL questionnaire for use in children aged 8–18 with repair of EA in and across 14 countries.

## 1. Introduction

**Figure 1:**
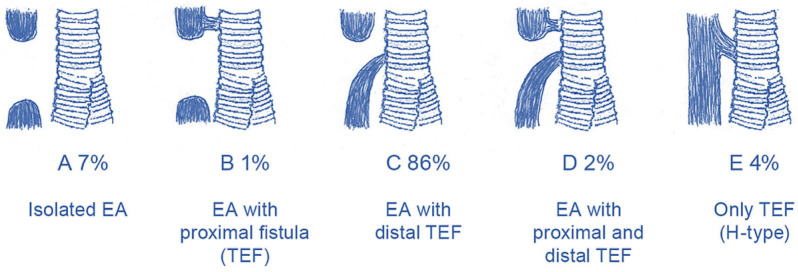
The Gross classification system of esophageal atresia and the prevalence of the different subtypes. This figure is included in this article with permission of Dr. Vladimir Gatzinsky.

Esophageal atresia (EA) is a rare congenital condition referring to an incomplete formation of the esophagus. There are different anatomical subtypes of EA with varying prevalence. EA is frequently associated with a tracheoesophageal fistula (TEF). Figure 1 provides an overview of the anatomical subtypes of EA according to the Gross classification system, including Gross A (isolated EA), Gross B (EA with proximal TEF), Gross C (EA with distal TEF), Gross D (EA with proximal and distal TEF) and Gross E (only TEF) [1]. EA also coincides with other anomalies, including the cardio-vascular (24–31%), uro-genital (19–21%), digestive (13–23%) and musculoskeletal (14–29%) [2,3]. Following Dr Cameron Haight’s first successful primary repair of EA in 1941 [4], the mortality rates of these children have decreased considerably, especially in the last decenniums [5]. Today, the vast majority of them undergo esophageal repair within the first days of life, with survival rates reaching around 90% [1]. This development has given rise to a new generation of survivors [6]. These children can be faced with long-term digestive morbidity (43–71%), including dysphagia [7], anastomotic strictures, gastroesophageal reflux disease [7,8,9], and feeding difficulties [10], as well as respiratory morbidity (52–69%), with wheezing, cough, dyspnea and recurrent respiratory infections [11,12]. The children risk growth retardation [13]; however, in affected children their growth may normalize at 12 years and beyond [14]. According to expert recommendations [15,16], health care providers of patients with EA should focus on optimizing their health-related quality of life (HRQOL). 

HRQOL is a multidimensional concept consisting of the physical, psychological and social health dimensions. By using generic and/or condition-specific HRQOL questionnaires, the patient’s perceived impact of disease and treatment can be evaluated [17]. The recent years reflect an increased number of HRQOL studies in children with EA [18,19]. The use of different generic HRQOL questionnaires has demonstrated varying outcomes in children with EA in comparison with the general pediatric population [20,21,22,23,24,25,26,27]. In the year 2018, a condition-specific measurement model for assessing HRQOL in children with EA aged 2–7 and 8–18 was completed (“The EA-QOL questionnaires”). The development of these questionnaires followed internationally well-established principles for patient-reported outcome measurements (PROMs) [28,29,30,31], meaning that the item generation was based on experiences told by children with EA and their parents in focus groups, and resulted in preliminary HRQOL questionnaires for children aged 2–7 and 8–18, respectively. In turn, the EA-QOL questionnaires’ psychometric properties were further evaluated in families of children with EA [32,33,34]. 

The respondent’s experience of the clarity, relevance and adequacy of an HRQOL questionnaire may be dependent on standards, norms and values employed in a country [29,35]. Such cultural characteristics may be integrated in the language spoken within a country, which is why language itself represents a main aspect to consider when establishing an HRQOL questionnaire internationally [35,36]. Therefore, well-established guidelines on principles for translation and cultural adaptation of a patient-reported outcome measurement should be used [37,38].

The EA-QOL questionnaires have been psychometrically evaluated in Swedish, German [34], Turkish [39], Polish [40] and Dutch [41] families of children with EA. During the past years, an increased number of countries have become engaged in the EA-QOL questionnaires. This could contribute to the benefit for children with EA from receiving a standardized condition-specific questionnaire to assess their HRQOL outcomes in research and clinical practice. Therefore, it was judged necessary to evaluate the EA-QOL questionnaires in multiple countries using a cross-cultural approach [42]. In order to enhance the understanding of the age-specific questionnaire versions of the measurement model, the study on the EA-QOL questionnaire for children aged 2–7 has been reported separately [43]. Although the methodological approach is similar, the results of the EA-QOL questionnaire for older children are unique, and as the measurement model consists of both versions the reported data are not complete until both cross-cultural evaluations are scientifically available for readers. Therefore, this study specifically aimed to describe an international collaborative initiative to establish a linguistic version of the EA-QOL questionnaire for children with EA aged 8–18 across 14 countries, prior to commencing an international field test.

## 2. Materials and Methods

### 2.1. Framework

**Figure 2:**
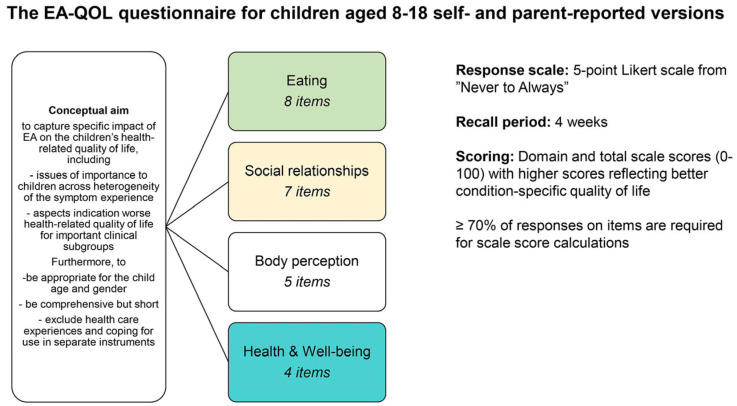
Presentation of the content, structure and aim of the EA-QOL questionnaire for children aged 8–18.

Figure 2 presents the EA-QOL questionnaire for children aged 8–18. The translation and cognitive debriefing of the EA-QOL questionnaire in a new country/language followed a standardized study protocol in English developed by the instrument developer (M.D.B.). The protocol employed international recommendations on PROMs [28,29,30,31,37,38], and aimed to support translations of the EA-QOL questionnaire to be conceptually equivalent and the implementation of a systematic psychometric evaluation across different countries.

### 2.2. Countries/Languages

All research teams had licensed the beforementioned study protocol and accepted participation in the international study group’s first joint initiative in 2021. These teams worked in Europe (Croatia, France, Germany, Hungary, Norway, Poland, Spain, Turkey, the United Kingdom (UK), Africa (South Africa), America (USA, Mexico) and Asia (China). In each of the country-specific research teams, there were experts in the field of EA who were native speakers and had received important insights into using the EA-QOL questionnaire in their setting. Furthermore, three patient representatives from the global support group for EA (EAT) accepted the invitation to ensure patient perspectives would be brought into the establishment of the EA-QOL questionnaire. 

### 2.3. Translation Procedure

**Figure 3:**
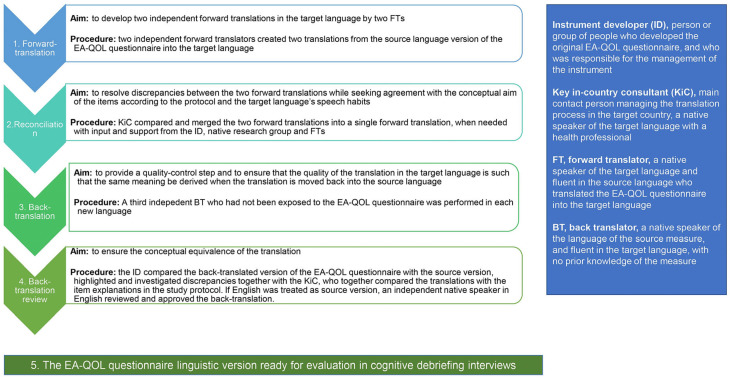
Methods used in the translation procedure of the EA-QOL questionnaire.

The forward–backward translation approach of the EA-QOL questionnaire is overviewed in Figure 3. After the establishment of the EA-QOL questionnaire in Sweden and Germany, the questionnaire was translated into 11 additional languages between 2017 and 2022, as described in more detail in Supplementary File S1. The translations worked as a basis for additional testing in 12 countries. 

### 2.4. Cognitive Debriefing

Cognitive debriefing interviews are used to gain information about how respondents experience and understand a questionnaire. Therefore, this method was used to make sure if and how the items of the EA-QOL questionnaire were adequate, understood in the way that instrument developers meant, and inclusive and complete, relative to the target concept (condition-specific HRQOL) and population (children with EA) [28,31,44]. As such, it aimed to maximize children’s and parents’ understanding of the translations and establish content validity. 

#### 2.4.1. Study Participants

Children aged 8–18 who were born with EA and one of their parents were invited to the cognitive debriefing interview. The goal was to recruit a small sample with varying severity of EA, as described in detail previously [40,41,45], and in agreement with the initial cognitive debriefing conducted in Sweden and Germany [33], as well as recommendations from the International Society for Pharmacoeconomics and Outcomes Research (ISPOR) [31,37]. Table 1 shows the characteristics of 82 children born with EA and 86 parents in 12 new countries who participated; 4–10 children and 5–10 parents participated in each country. In the cross-cultural sample, 80.2% of the children who gave self-reports were born with Gross EA type C, 62.2% were male, and the majority of parent-reports came from mothers (81.4%). 

#### 2.4.2. Data Collection

**Figure 4:**
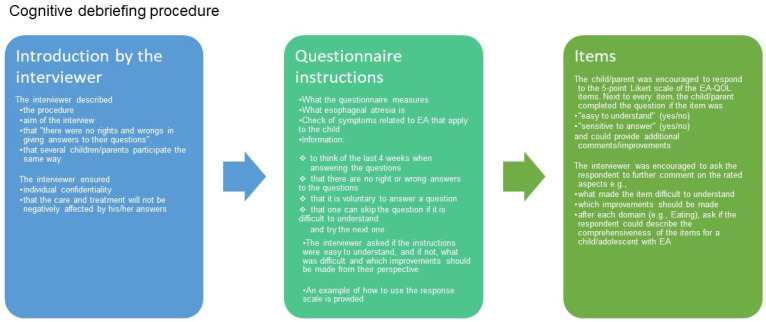
The cognitive debriefing procedure of the EA-QOL questionnaire.

In Supplementary File S2 the method of collecting data in the cognitive debriefing interviews of the EA-QOL questionnaire for children aged 8–18 can be read in greater detail. Mostly, the study participants were recruited from clinical centers only *(n* = 9 countries), in two countries (Norway and Hungary) from patient support groups and clinical centers, and in one country (the UK), from a patient support group (TOFS). In ten countries, the participants were interviewed at the clinical center/hospital and by a researcher in the health care profession. All children and their parents were interviewed separately. Figure 4 outlines the cognitive debriefing procedure. During the interviews, notes of children’s and/or their parents’ comments on the questionnaire were taken and documented in their own language. The key in-country person (national study coordinator) translated these into English and registered them in a standardized Excel file for documentation and sent them to the instrument developer (M.D.B.). Lastly, the instrument developer merged all data into one cross-cultural database file. 

#### 2.4.3. Data Analysis

Statistical data were analyzed using IBM SPSS Statistics for Windows (Version 25.0, Armonk, NY, USA: IBM Corp). Descriptive statistics were used to analyze study participant characteristics as well as the items in the EA-QOL questionnaire (self- and parent-report), considering item clarity (yes/no), sensitive/uncomfortable to answer (yes/no), and missing item responses (*n*, %), on a country-specific level, and accumulated cross-cultural level. Data from Sweden and Germany [33] were considered to be a framework for a primary evaluation of the items. Therefore, these data were excluded in the cross-cultural analysis of the translated items evaluated in the 12 new countries.

First, children’s and parents’ comments were sorted by a country-specific researcher into negative/difficult and confirmative experiences of each item. Data from all 12 countries were then analyzed using a manifest content analysis to define the categories of the strengths and difficulties of the translated items. A respondent’s statement regarding an item could only belong to and be sorted into one category [46]. The suggested categories from each country were reviewed and discussed with all native research teams. Following their input, all cross-cultural categorizations were proposed. Together with two researchers (JHQ, SW), these were evaluated and completed. Descriptive statistics of the number of respondents making a comment were analyzed in relation to each category. Additionally, their experiences on other questionnaire components (the response scale and questionnaire instructions) were compiled. 

**Figure 5:**
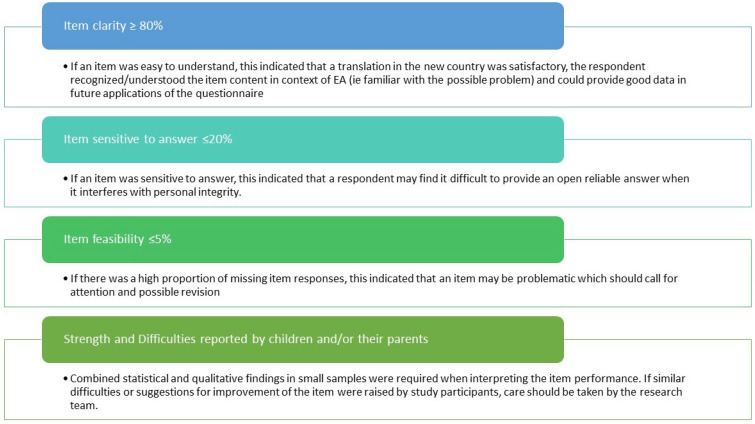
Predefined psychometric criteria applied in the analysis of the newly translated EA-QOL questionnaires.

The quantitative and qualitative information was analyzed against predefined psychometric criteria also applied in the initial analysis of the performance of the EA-QOL questionnaires [33,34], as presented in Figure 5. These criteria were used to indicate the need for adjusting and rewording the item in order to better achieve linguistic, cultural and conceptual adequacy in the specific country/language. 

### 2.5. Harmonization between Different Language Versions of the EA-QOL Questionnaire

The harmonization aimed to achieve equivalence between the Swedish version, the target-language version and all translations of the EA-QOL questionnaire from a conceptual point of view. The focus was therefore to identify any translation discrepancies that occurred between them and revise the translations congruently with each other [37,38]. The harmonization could occur at any point during the translation process and always involved the instrument developer (M.D.B.) who had been supporting the development of each translation. In the USA, the United Kingdom and South Africa English was spoken and in Spain and Mexico, Spanish. In the same languages employed in several countries the translations were compared and harmonized after the cognitive debriefing with the support of native professionals/researchers (C.d.V., B.Z., S.E., N.D., A.S.G., J.D.H.P.), an instrument developer (M.D.B.), and a patient representative (GS). Moreover, the Chinese Mandarin and Hungarian translations were compared and reviewed with the English version of the EA-QOL questionnaire by the native experts (Hungary K.M. and China S.L.) and the instrument developer (M.D.B.).

### 2.6. Deciscion on the Need to Change the Translations

The decision on the need to modify item wording was made based on native expert and instrument-developer review, cognitive debriefing results, input from patient stakeholders and harmonization needs between different language versions. The children’s self-report was regarded as of primary importance [29]. The international item performance provided indications for making the changes in the EA-QOL questionnaire cross-culturally and was decided by the original instrument developers (MDB, JQ, SW, and JD), after receiving input from the international author group.

## 3. Results

Supplementary File S3 presents findings from the cognitive debriefing of the 24 translated items in the EA-QOL questionnaire for children with EA conducted with participants from (in alphabetic order) China, Croatia, France, Germany, Hungary, Mexico, Norway, Poland, South Africa, Spain, Turkey, the United Kingdom (UK), and the USA, and Table 2 displays the cross-cultural number and percentage of respondents who rated the item as clear (easy to understand), sensitive/uncomfortable to answer, and the number and proportion of missing item responses.

### 3.1. Item Clarity

Similar to the study findings in Sweden and Germany [33], 100% of the items in the cross-cultural analysis of data from 12 countries achieved the criteria for clarity ≥80% in self- and parent-report (Supplementary File S3). In the self-report, ≥95% of the children in all countries rated all items easy to understand except for one case (one out of four children from Mexico did not rate item 2 as easy to understand), and in the parent-report all translated items achieved the criteria regarding its clarity in 8/12 countries. In the four additional countries, <80% of the parents rated the following items as easy to understand, and therefore not achieving the desirable criteria: Spain (item 8) and Croatia (items 22–24), Norway (items 1, 5, 14, 15, 23) and the UK (items 4–8, 14, and 19).

### 3.2. Item Sensitive/Uncomfortable to Answer

Like the study findings in Sweden and Germany [33], the cross-cultural analysis showed that all items for children aged 8–18 achieved the criteria for sensitive/uncomfortable to answer in self- and parent-report (Supplementary File S3). However, variation was observed between individual countries.

None of the children from the six countries of Croatia, Poland, Hungary, Spain, Mexico, and China rated any of the items as sensitive/uncomfortable to answer. In the US and South Africa, 2/6 children (33%) rated seven items as sensitive/uncomfortable to answer—these mainly concerned information about their body perception (USA) or social relationships (South Africa). Cross-culturally, the item most commonly rated as sensitive/uncomfortable to answer by children asked the child about their experiences of being sad due to EA (item 24). This item was rated sensitive/uncomfortable by >20% among children from the UK (*n* = 4), France (*n* = 1), and South Africa (*n* = 2).

None of the parents from Croatia, Poland, Spain, France, Mexico, or China rated the items as sensitive/uncomfortable to answer, but more than 20% of parents from UK rated two items which regarded experiences of social exclusion (item 11 and item 14) and two items which regarded surgical scars (item 18, item 19) as sensitive/uncomfortable to answer. Similarly > 20% of parents from Norway rated one item concerning experiences of social exclusion (item 14) and body perception (items 18, 19 and 20) as sensitive/uncomfortable to answer. Furthermore, in South Africa, four items were rated as sensitive/uncomfortable to answer by 2/6 (33%) of parents and these related to social relationships (item 9 and item 11), surgical scars (item 19), and breathing difficulties (item 21). The item most commonly rated as sensitive/uncomfortable to answer by parents considered their children’s looks due to surgical scars (item 19). 

### 3.3. Item Feasibility

As in study results from Sweden and Germany [33], all items achieved the criteria for item feasibility of missing item responses ≤5% in self- and parent-report.

### 3.4. Children’s and Parents’s Comments

**Figure 6:**
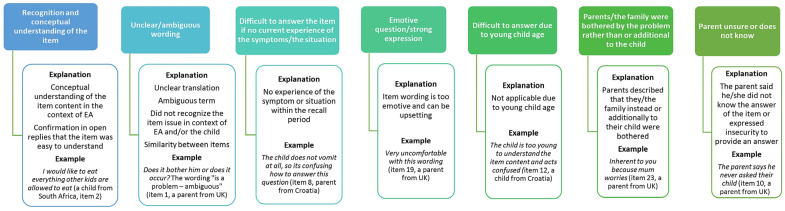
Categories of the strength and difficulties of the translated items included in the EA-QOL questionnaire for children aged 8–18 based on content analysis of children’s and parents’ comments during the cognitive debriefing.

Supplementary File S4 shows the categories of children’s and parents’ comments on the items in the EA-QOL questionnaire. Comments revealing the understanding/recognition of the item were given by a sub sample (maximum comments from 12 children/10 parents), and those revealing difficulties of an item were also given by a sub sample (maximum comments from 9 children/13 parents). The explanation of the strength and difficulties categories are overviewed in Figure 6.

#### 3.4.1. Categories of Children’s and Parents’ Comments

All items received at least one comment from children and/or their parents, which illustrated their understanding of the content/issue displayed in the item and/or confirmation in open replies that the item was clear. 

Four categories of perceived difficulties of the items were identified, as listed below. Generally, the comments concerned unclear/ambiguous wording.

- Unclear/ambiguous wording: across 2–4 countries, four items within the eating domain asking about the child’s experiences of food getting stuck (item 1), choking (item 5–6), and vomiting (item 8) received comments from ≥5 children and/or parents that considered unclear/ambiguous wording. These comments gave explanations as to why parents in Norway and the UK rated some items “not easy to understand”. Parents in China wished for clarification of the idea that food gets stuck in the esophagus, not the throat (item 1). Parents in three countries (China, the UK and Hungary), also asked about the definition of “choke” (item 5–6), to ensure it referred to “cough caused by inhalation of food into the trachea while eating” [45]. The parents from the UK described the fact that two original items needed clarification as to whether the child was bothered by the symptom or only if the symptom was present in the child (items 1 and 8), and asked if vomiting (item 8) included regurgitation;
- The category in which it was difficult to answer the item without current experience of the situation mainly concerned the child’s communication about EA with other people, and these comments were mentioned by five children from three countries (item 10) and five children from five countries (item 15);
- Emotive question/strong expression: in agreement with the rating of items as sensitive/uncomfortable to answer, parents from the UK commented that items asking about the child’s social relationship (item 14, in which the translation originally used the word “nasty” instead of “unkind”) and self-perception due to surgical scars (item 19, in which the translation originally used the word “feeling less perfect”) were emotive in the English language;
- Difficult to answer due to young child age: these comments regarded four items (items 9–12) given by one child from Croatia, and four items (items 21–24) commented on by one parent from Croatia.

The two additional categories (Figure 6) included very few comments (Supplementary File S3).

#### 3.4.2. Item Comprehensiveness

Three aspects that related to the item comprehensiveness were mentioned. The EA-QOL questionnaire did not include questions about HRQOL issues related to associated anomalies (Norway). Moreover, participants wished that the items would better address physical activity/physical performance (Norway) and the family’s eating habits (UK).

#### 3.4.3. Questionnaire Instructions

Respondents from one country (Norway) stated that the questionnaire instructions were too extensive and difficult.

#### 3.4.4. Response Scale

In the UK, Norway, Croatia, Hungary, and the US, the “non-applicable” response option was proposed by study participants or experts. One child from the US wanted a numerical value for the ordinal scale never-to-always.

### 3.5. Harmonization

Table 3 lists which translated items of the EA-QOL questionnaire for children aged 8–18 did not achieve the desired statistical psychometric criteria and which were reworded to improve its clarity/adequacy in the individual countries/languages. As shown, in five countries (Croatia, France, Hungary, Poland, and Turkey), no changes in the wording of the translated items were judged to be needed. Altogether, most improvements in the translated items were conducted in the domain eating. A detailed description of the process for individual countries is included in Supplementary File S5. A linguistically equivalent UK- and US-English version and a linguistically equivalent European Spanish–Mexican Spanish version of the EA-QOL questionnaire, respectively, were established. No cross-cultural change in the EA-QOL questionnaire was needed.

## 4. Discussion

This study describes an international collaborative initiative to establish an appropriate linguistic version of the EA-QOL questionnaire for children with EA aged 8–18 in 12 additional countries from different continents, following its development in Sweden and Germany. It completes the evaluation of the whole measurement model EA-QOL, following an evaluation of the age-specific version for 2–7-year-old children. 

### 4.1. Translation

This study has evolved gradually from items generated in Sweden [32], a country in the northern part of Europe with a language spoken by around ten million people [47]. Although Sweden is among the countries where most PROM studies in pediatric surgery are conducted [42], HRQOL questionnaires have mostly been developed in the English language [48,49]. Following the EA-QOL questionnaire’s finalization in Sweden and Germany [34], 12 further translations were established with the help of forward and backward translators, native specialist teams, and instrument developer(s). As noted, this step requires investment in both competence and time [50], but care is needed to achieve a correct conceptualization of a measurement across languages/countries. This in turn permits future pooling of international data sets and creates benefits from increased sample size in future research [37,50], which is desirable in a rare condition like EA [19,42].

The translation process of the EA-QOL questionnaires was guided by the ISPOR principles [37]. Complete compliance with these principles may be difficult for PROMs which are being internationally adapted [51], and, for example, we did not only work with professional translators, which may influence the findings. However, we gave emphasis to some key components. One of them was the close collaboration with a key in-country person and the instrument developer. Another key component was a study protocol with explanations of the items [37]. Nevertheless, because of resources and availability of bilingual translators, the translation procedure in our study varied between countries concerning, for example, the time point of the translation and recruitment source for translators. As recommended for HRQOL instruments [37], we aimed to establish conceptual equivalence with the Swedish source version and the new translations of the EA-QOL questionnaire [35,37], as otherwise the instrument will be less likely to demonstrate the validity and reliability that the original version did [37]. Already in the back-translation review, between 2 and 10 items were improved with respect to the wording in the child-report and 5–10 items in the parent-report in order to reach semantic equivalence (Supplementary File S1), with efforts made to achieve semantic agreement with the original version and equivalence between the self- and parent-report [51,52]. 

### 4.2. Cognitive Debriefing

In this study, the vast majority of the patients themselves rated the translated items of the EA-QOL questionnaire as easy to understand. In HRQOL research, the self-report is of primary importance [29], and underlines the significance of these findings. However, the aim of the cognitive debriefings was also to identify poor translations, improve them, and try to optimize the understanding of the items from both children’s and parents’ perspectives [31]. Therefore, in our study, the parents’ views of poorly translated items was helpful in achieving this goal. 

According to our cross-cultural results, 18/24 items in the self-report and 19/24 items in the parent-report were judged as sensitive/uncomfortable to answer by less than 5% of the study participants. However, in a few countries, some items were felt to be sensitive/uncomfortable by a few participants and, interestingly, these items frequently concerned questions about a child’s social exclusion due to EA and surgical scar(s). In the Swedish–German focus groups, these aspects were clearly reported as important issues for HRQOL in children with EA and experiences of social exclusion seemed to affect a severely affected subgroup [32,53]. The items being perceived as sensitive/uncomfortable to answer could indicate that the denotation in the new language was too emotive, suggesting the need to modify the item wording. For example, this was the reason for improving the item wording related to social exclusion and surgical scars in the English language. Moreover, the perception of a question being sensitive could indicate how comfortable the child was in discussing these aspects of their health or the impact on their mental health. A child’s perception of a question being sensitive to answer may in turn be related to the child’s individual (personality) characteristics, the family’s communication style about the child’s condition, the availability for them to meet peers in a support group for EA, the health care provider’s communication in a patient encounter, and cultural norms. Cross-culturally, the most sensitive/uncomfortable question, rated as such by 9.9% of the children, asked if they experienced sadness due to EA. Interestingly, some children and parents also described that even though a question is sensitive/uncomfortable to answer for them, it may still be important to ask, which underlines the need for caring for both physical and mental health. In this study, many cognitive debriefing interviews were conducted at the outpatient clinic service with a researcher who had a health-care professional background. In previous research, the implementation of HRQOL questionnaires into clinical practice with children with chronic conditions has shown to increase the focus on psychosocial issues in the communication between the health care provider and the patient and the children’s insights into their health [54]. The sample sizes in individual countries were low and, commonly, the “same” individuals reported several items as sensitive/uncomfortable to answer. For example, two out of six children from South Africa rated seven items sensitive/uncomfortable to answer. However, in a subsequent pilot study from South Africa utilizing the EA-QOL questionnaire, the results proposed it to be a valuable part of follow-up care, especially for children aged 8–18 giving their self-report [55]. The respondents’ perception of a PROM is important and has also been evaluated in another study using the condition-specific CLEFT-Q among persons aged 8–29 with cleft lip and/or palate. The authors found that after completing the questionnaire 23% of the respondents reported feeling upset or unhappy about their appearance or how they look, and this was associated with reporting lower CLEFT-Q-scores (i.e., worse outcomes), female gender, severity of the cleft, anticipating future cleft-specific surgeries, and country of residence [56].

Whereas the instructions of the EA-QOL questionnaire had very few complaints, we discussed if the response option “not applicable” could be helpful and make the questionnaire better adapted to children with EA, who represent a population with varying presentation in symptomatology. The recommendations for PROMs used in the development of the EA-QOL questionnaires described how the use of such a response option could lead to bias in the scoring [28] and can cause problems [57], because it can provide the respondent with an option to not reply if they are uncertain of a question or not willing to answer [58,59,60,61]. Difficulties in interpreting what the “not applicable” stands for may arise [57]. If it is treated statistically as “never a problem” its use could bias the results towards “not affected” [28]; however, the risk of bias remains if these patients reply “never a problem” but have never experienced the situation which is asked about. We did, however, ensure that questionnaire instructions of the EA-QOL questionnaires in all languages stated that the respondent may skip an item, e.g., when an item is difficult to understand and/or does not apply to them. In the EA-QOL questionnaires, the respondent should complete 70% or more of the items in order for them to be included in the scale score calculations [34]. In comparison, well-established instruments like PedsQL [62] or CLEFT-Q [63] recommend ≥50% of the item responses should be answered. Furthermore, the EA-QOL items were evaluated for missing item responses, a well-established method for identifying problematic items [64], and our study demonstrated excellent results for the completeness of data. Moreover, heterogeneity of a condition means that the inclusion criteria for a PROM’s target population is essential. Similar to instruments assessing outcomes related to peroral eating/feeding [65,66], the eating domain of the EA-QOL questionnaire becomes less applicable for children who are solely fed enterally. It is important to recognize that children who are tube-fed have special and additional needs related to physical health and emotional well-being, but also to the social exclusion that comes from situations where food plays a significant role [67]. This could underline the need for using the last section of the EA-QOL questionnaires, where children and parents “can write things that we have not asked you about” to enable their experiences to be considered. However, this cognitive debriefing study showed that the EA-QOL questionnaire was comprehensive from the perspectives of children and parents, which is important for its content validity [30,31]. A limitation of the EA-QOL questionnaires is that they do not include questions about how associated anomalies may influence the child’s HRQOL, as these items did not perform sufficiently well in the initial psychometric tests [32,33].

Our study reflects an evaluation of the EA-QOL questionnaire in 12 further countries, following its finalization in Sweden and Germany. In comparison, a simultaneous cross-cultural development of a PROM in multiple countries could increase the possibilities of international applicability [68,69,70]. Harmonizing the translations of a PROM is central to achieve intertranslation validity [37,38]. In order to optimize the intertranslational validity, we coordinated the experiences of 14 translations of the EA-QOL questionnaire, including for the same languages employed in different countries [38]. For these languages we decided to perform the harmonization after the cognitive debriefing in the respective countries. Interestingly, in earlier studies [71,72,73,74], the cross-cultural harmonization process of HRQOL instruments in children of different languages has been performed in varying ways. 

### 4.3. Study Strengths and Weaknesses

A strength of the current study is the international collaboration between instrument developers, patient stakeholders and native experts in the field of EA in 14 countries, as well as the inclusion of the perspectives of families of children with EA. 

Despite applying a standardized study protocol, the study is limited by variation in the translation procedure, data collection, recruitment sources, and experiences of the interviewer. The reasons for such variations are each study center’s/country’s prerequisites for carrying out the study, including available competence, resources and sample sizes. In individual countries the study samples were small but generally in agreement with recommendations for cognitive debriefing [37,44]. There is also a lack of clinical data in this report, which impedes the understanding of the study findings. We described only the participating children’s subtypes of EA. The prevalence of Gross C seems slightly lower and that of Gross A higher (16% in self-report) compared with other reports [1]. This may be a result of the fact that we used purposive and convenient sampling methods stratifying for the severity of EA, and that many of our study centers are health care providers of highly specialist care for children with EA in their countries. Still, the case load of EA and the experience of follow-up after reconstructive surgery may vary between the study centers, which may influence the findings.

Furthermore, a study strength is our combined qualitative and quantitative approach in evaluating the EA-QOL questionnaire. In qualitative research, however, one informant’s comment could be equally important as comments made by several informants [75], which should be considered in relation to the quantification of parents’ comments. However, the differences in the number of interview data between the countries limits the study, and may be explained by the quality of the translations and/or the interviewer competence. Moreover, this study did not focus on differences in socio-economic cultures within the same country, the patients’ or parents’ educational level, or their mental or cognitive functioning.

It may also be seen as a study weakness, that the cross-cultural work on the questionnaire version for children aged 2–7 has been reported separately [43]. However, this approach was deemed necessary as the results are unique to each age-specific questionnaire and the extent of data presented is large. Furthermore, this study completes the cross-cultural approach for evaluating the measurement model EA-QOL. The items included in the EA-QOL questionnaires have not yet been evaluated regarding differential item functioning (differences in the functioning of items across groups) between children with EA from different country cultures [36]. There is a discussion in the literature regarding whether requirements of cross-cultural equivalence of an HRQOL questionnaire can be fulfilled or not [35,48]. On one hand, experiences of diseases may be related to culture, and on the other hand the concept of HRQOL may have universal components across cultures. Future research should therefore include a multi-center international field test of the EA-QOL questionnaire with a larger sample size, which would enable the use of differential item functioning to determine whether different subgroups of child age, sex and country of residence respond differently to items within the EA-QOL questionnaires. Furthermore, the cross-culturally valid and reliable versions should be used to understand more about risk groups for impaired condition-specific HRQOL and profiles of impacted HRQOL domains related to child age, gender, surgical treatment and country of residence in cross-sectional as well as longitudinal study designs. It will be important to investigate the children’s and parents’ perception of answering the EA-QOL questionnaires as part of follow-up care, the time required to complete them and the effect of implementing the EA-QOL questionnaire into routine clinical care, and, moreover, to better address which factors are associated with children’s and parents’ positive and negative experiences of completing the EA-QOL questionnaire. In the end, such study results can help inform parent/patient education and care pathways and provide pointers to areas which might require further research. The work of establishing a cross-cultural disease-specific questionnaire for a rare and complex pediatric surgical condition like EA has offered important methodological experiences. This study can potentially benefit future research within this field when researchers are considering and preparing a development of a disease-specific questionnaire for other gastrointestinal conditions in pediatric surgery.

## 5. Conclusions

Through international collaboration, a conceptually and semantically equivalent HRQOL questionnaire for children with EA aged 8–18 has been established for use in 14 countries, supporting the EA-QOL questionnaire’s content and linguistic validity with no need for any conceptual cross-cultural change. This international collaborative approach was needed to resolve poor translations or lack of cultural appropriateness of individual language translations. After an international field test of the EA-QOL questionnaire’s psychometric properties in families of children with EA aged 8–18, this study will help researchers, health care providers and patient stakeholders to enter the next era in outcome research, but also in the clinical context of use regarding children with EA. 

## Figures and Tables

**Table 1:** Characteristics of the study sample in 12 new countries.

	*n* (%), Self-Report (*n* = 82)	*n* (%), Parent-Proxy Report (*n* = 86)
Child male sex	51 (62.2)	55 (64.0)
Gross type ^a^		
A, isolated EA	13 (16.0)	14 (16.7)
C, EA with distal TEF	65 (80.2)	67 (79.8)
D, EA with prximal and distal TEF	2 (2.5)	2 (2.4)
E, only TEF	1 (1.2)	1 (1.2)
Child age (median/min–max)	12 (8–18)	11 (8–18)
Country of residence		
United Kingdom	10 (12.2)	10 (11.6)
Spain	9 (11.0)	9 (10.5)
Poland	9 (11.0)	9 (10.5)
Croatia	9 (11.0)	9 (10.5)
Norway	8 (9.8)	8 (9.3)
Hungary	6 (7.3)	6 (7.0)
USA	6 (7.3)	6 (7.0)
China	6 (7.3)	6 (7.0)
South Africa	6 (7.3)	6 (7.0)
Turkey	5 (6.1)	7 (8.1)
Mexico	4 (4.9)	5 (5.8)
France	4 (4.9)	5 (5.8)
Parent-proxy mother		70 (81.4)
Parent-proxy age (median/min–max)		43 (26–66) ^b^

^a^ one missing value in a self-report EA, esophageal atresia; TEF, tracheoesophageal fistula; ^b^ four missing values in parent-proxy reports.

**Table 2:** Findings from the cognitive debriefing with parents of 82 children born with esophageal atresia aged 8–18 and 86 parent-proxies from 12 countries.

		Easy to Understand ^a^		Sensitive/Uncomfortable to Answer ^a^		Missing Item Responses ^b^	
		Self-Report	Parent-Proxy Report	Self-Report	Parent-Proxy Report	Self-Report	Parent-Proxy Report
	**Items—Eating**						
1.	I feel distressed that food gets stuck in my throat when I eat	81 (98.8)	81 (94.2)	3 (3.7)	0	0	0
2	My health condition restricts me from eating certain foods’	79 (96.3)	83 (96.5)	1 (1.2)	1 (1.2)	1 (1.4) ^c^	1 (1.4) ^d^
3	It hurts when I eat because of my health condition (e.g., when food sticks, heartburn, tummy ache)	81 (98.8)	84 (97.4)	1 (1.2)	1 (1.2)	0	2 (2.7) ^d^
4.	I have to remind myself to drink liquids when I eat	80 (97.6)	80 (93.0)	2 (2.4)	2 (2.3)	0	0
5	I am afraid when I choke while eating	79 (96.3)	81 (94.2)	2 (2.4)	2 (2.3)	0	0
6.	I feel that my experiences of choking make it difficult for me to eat	79 (96.3)	81 (94.2)	1 (1.2)	1 (1.2)	1 (1.4) ^c^	0
7	I can eat at the same speed/pace as other children my age	80 (97.6)	83 (96.5)	1 (1.2)	2 (2.3)	0	0
8.	It bothers me if I vomit after I eat	79 (96.3)	74 (86.0)	1 (1.2)	3 (3.5)	0	0
	**Items—Social relationship**						
9.	I feel like the only one who was born with esophageal atresia	79 (96.3)	85 (98.8)	5 (6.1)	6 (7.0)	0	0
10.	It is complicated to explain to others what esophageal atresia is	79 (96.3)	82 (96.5) ^a^	1 (1.2)	1 (1.2)	0	0
11.	People call me names (perhaps because of your size, having an unusual cough, eating slowly, or because you have a surgical scar)	79 (96.3)	85 (98.8)	5 (6.1)	8 (9.3)	0	0
12.	I feel that other people are staring at me (e.g., when coughing, choking, dressing in the locker room)	81 (98.8)	86 (100)	6 (7.3)	2 (2.3)	0	0
13.	I get tired of people asking about the scar/scars	81 (98.8)	86 (100)	3 (3.7)	1 (1.2) ^a^	0	0
14.	Other people say unkind things about me	79 (96.3)	81 (94.2)	2 (2.5) ^a^	8 (9.3)	0	1 (1.4) ^d^
15.	It feels awkward when other people ask me about esophageal atresia	78 (95.1)	84 (97.7)	2 (2.4)	3 (3.5) ^a^	0	0
	**Items—Body Perception**						
16.	I feel different because I have scars	80 (97.6)	85 (98.8)	4 (4.9)	4 (4.7)	0	0
17.	I am careful about what I wear because of my scar/scars	78 (96.3) ^a^	85 (98.8)	5 (6.2) ^a^	2 (2.3)	0	0
18.	I feel awkward when my scar/scars are visible to other people (e.g., new people, boy- or girlfriend, people in the changing room, or in the swimming pool)	80 (98.8)	83 (96.5)	4 (4.9) ^a^	6 (7.0)	0	1 (1.4) ^d^
19.	I am unhappy with the way I look because I have scars	78 (97.5) ^b^	81 (95.3) ^a^	7 (8.8) ^b^	10 (11.6)	1 (1.4) ^c^	1 (1.4) ^d^
20.	It bothers me that I am smaller than children my age	80 (98.8) ^a^	85 (98.8)	4 (4.9) ^a^	2 (2.3)	0	0
	**Items—Health and Well-being**						
21.	I am bothered by breathing difficulties if I exercise and play	80 (98.8) ^a^	84 (97.7)	3 (3.7) ^a^	2 (2.3)	0	0
22.	I have trouble falling or staying asleep at night because of my health condition (e.g., acid reflux, heartburn, or respiratory problems)	80 (98.8) ^a^	82 (95.3)	1 (1.2) ^a^	2 (2.3)	0	0
23.	I am worried about my future because of esophageal atresia (e.g., school, friends, boy- or girlfriend, work)	80 (98.8) ^a^	82 (95.3)	3 (3.8) ^b^	4 (4.7)	0	1 (1.4) ^d^
24.	Esophageal atresia makes me sad	81 (100) ^a^	82 (95.3)	8 (9.9) ^a^	3 (3.5)	0	0

^a^ Children from Croatia, France, Hungary, Norway, Poland, Spain, Turkey, the United Kingdom, the USA, Mexico, China, South Africa; ^b^ excluding data from Sweden, Germany, Norway and China, therefore inclusion of 69 self-reports and 73 parent-proxy reports; ^c^ one child from Spain did not provide an answer to item 2 or item 6 and one child from the UK skipped item 19; ^d^ one parent from the United Kingdom did not provide answers to items number 14 or 19, one parent from Mexico to item number 2, two parents from France did not answer item number 3, and one parent from France did not answer the items numbered 18 and 23.

**Table 3:** Overview of translated items of the EA-QOL questionnaire for children aged 8–18 (child-report, C; parent-report, P) which did not fulfill the desired criteria and which items were changed with respect to item wording.

	Items not Fulfilling the Desired Criteria					Modification/Changes in Item Wording			
Language	Item Clarity ^a^	Item Sensitive to Answer ^b^	Item Feasibility ^c^	No Change	Response Scale	Eating (Items 1–8)	Social Relationship (Items 9–15)	Body Perception (Items 16–20)	Health and Well-Being (Items 21–24)
Turkish				X					
Polish				X					
Hungarian				X					
Croatian	P: 22, 23, 24			X					
French		C: 24		X					
Norwegian	P:1, 5 14, 15, 23	P: 14, 18, 19, 20				1, 5, 6			
Chinese						1, 5, 6			
UK English	P: 4, 5, 6, 7, 8, 14, 19	C: 24			Was seldom changed, to rarely	1, 2, 4, 5, 6, 7, 8	14	19	22
		P: 11,14, 18, 19							
US English		C: 16, 17, 18, 19, 20, 21, 23			Was seldom changed, to rarely	1, 2, 4, 5, 6, 7, 8	14	19	22
South African English		C: 9, 11, 12, 16, 17, 24			Was seldom changed, to rarely	1, 2, 4–8	14, 15	17, 18, 19, 20	22
		P: 9, 11, 19, 21							
European Spanish	P: 8					1, 3, 4, 5, 6, 8	10, 11, 14	16, 17, 18	21, 23, 24
Mexican Spanish	C: 2					1, 2, 3, 4, 5, 6, 8	10, 11, 12, 15	16, 17, 18, 19, 20	21, 22, 23, 24

^a^ Item clarity ≥80%, ^b^ Item sensitive to answer ≤20%, ^c^ Item feasibility ≤5%, including data from Croatia, France, Hungary, Poland, Spain, Turkey, the United Kingdom, South Africa, Mexico and the USA; i.e., data from Norway and China were excluded in this study.

## Data Availability

The datasets analyzed during the current study are available in the manuscript or in its Supplementary Files. Further information is not available in public due to lack of ethical approval.

## Supplementary Materials

The following supporting information can be downloaded at: https://www.mdpi.com/article/10.3390/children11030286/s1, Supplementary File S1: The target languages, year of establishment, key in-country consultants, professionals involved in the forward–backward translation, and result of the back-translation review; Supplementary File S2: Description of the data collection method used in the cognitive debriefing interviews of the EA-QOL questionnaire for children aged 8–18 listed in order of the earliest study year and country; Supplementary File S3: Description of the number and percentage of children with esophageal atresia aged 8–18 and their parent-proxies who rated the items of the EA-QOL questionnaire as easy to understand in Sweden–Germany as a reference from the primary item evaluation and for Europe (Croatia, France, Hungary, Norway, Poland, Spain, Turkey, United Kingdom, Africa (South Africa), Asia (China), Central America (Mexico) and North-America (USA) as the new item evaluation; Supplementary File S4: Categorization of children’s (4a) and parents’ (4b) comments of the understanding and perceived difficulties of items in the EA-QOL questionnaire for children with esophageal atresia aged 8–18; Supplementary File S5: Implications of cognitive debriefing, expert review and harmonization of the EA-QOL questionnaires for children aged 8–18 in individual countries.

## Appendix A

The following authors are part of “The international EA-QOL group”.

Michaela Dellenmark Blom ^1,2,3,^*, Stefanie Witt ^4^, Natalie Durkin ^5^, Simon Eaton ^5^, Alba Sánchez Galán ^6^, Anna Rozensztrauch ^7^, Ivana Sabolić ^8^, Kjersti Birketvedt ^9^, Benjamin Zendejas ^10^, Katalin Eszter Müller ^11,12,13^, Siqi Li ^14^, Corné de Vos ^15^, Tutku Soyer ^16^, Juan Domingo Porras-Hernandez ^17^, Anastasia Fourtaka ^18^, Graham Slater ^19^, Ragnhild Emblem ^20^, John Bennett ^10^, Robert Smigiel ^7^, Darius Patkowski ^21^, Ana Špoljarić ^8^, Marina Stilinović ^8^, Zita Andrásdi ^11^, Dora Škrljak Šoša ^8^, Tomislav Luetić ^8^, Sylwester Gerus ^21^, Çiğdem Ulukaya Durakbaşa ^22^, Jinshi Huang ^14,23^, Shen Yang ^14^, Yong Zhao ^14^, Yichao Gu ^14^, Shuangshuang Li ^14^, Miram Pasini ^8^, Frederic Gottrand ^18^, Orsolya Kadenczki ^24^, Leopoldo Martínez Martínez ^6^, Vuokko Wallace ^19,25^, Anke Widenmann ^19^, Feliciana Milagres Sikwete ^15^, Diego Rodriguez-Alvirde ^17^, Shawn Izadi ^10^, Benno M. Ure ^26^, Daniel Sidler ^15^, Jens Dingemann ^26,‡^ and Julia H. Quitmann ^4,27,‡^

### Appendix A.1. Affiliations

1 The Department of Pediatrics, Institute of Clinical Sciences, University of Gothenburg, 40530 Gothenburg, Sweden
2 The Department of Pediatric Surgery, Queen Silvia Children’s Hospital, 41685 Gothenburg, Sweden
3 Department of Women’s and Children’s Health, Karolinska Institutet, 17177, Stockholm, Sweden
4 Department of Medical Psychology, University Medical Center Hamburg-Eppendorf, 20251 Hamburg, Germany
5 UCL Great Ormond Street Institute of Child Health, 30 Guilford Street, London WC1N1EH, UK
6 Department of Pediatric Surgery, University Hospital La Paz, 28046 Madrid, Spain
7 Department of Nursing and Obstetrics, Division of Family and Pediatric Nursing, Wroclaw Medical University, 50367 Wroclaw, Poland
8 Division of Pediatric Surgery, Department of Surgery, University Hospital Centre Zagreb, 10000 Zagreb, Croatia
9 Centre for Rare Disorders, Oslo University Hospital, Rikshospitalet, 0372 Oslo, Norway
10 Esophageal and Airway Treatment Center, Department of Pediatric General Surgery, Boston Children’s Hospital, Harvard Medical School, Boston, MA 02115, USA
11 Heim Pál National Pediatric Institute, 1089 Budapest, Hungary
12 Institute for Translational Medicine, Faculty of Medicine, University of Pécs, 7622 Pecs, Hungary
13 Doctoral School of Clinical Medicine, University of Debrecen, 4032 Debrecen, Hungary
14 Department of Neonatal Surgery, Beijing Children’s Hospital, Capital Medical University, National Center for Children’s Health, Beijing 100045, China
15 Tygerberg Hospital, Stellenbosch University, Stellenbosch 7602, South Africa
16 Department of Pediatric Surgery, Faculty of Medicine, Hacettepe University, 06100 Ankara, Turkey
17 Surgical Division, Hospital para El Niño Poblano, Puebla 72000, Mexico
18 Centre Hospitalier Universitaire Hôpital Jeanne de Flandre Pôle Enfant 2, Avenue Oscar Lambret, 59037 Lille CEDEX, France
19 EAT (Esophageal Atresia Global Support Groups), Sommerrainstr. 61, 70374 Stuttgart, Germany
20 Department of Paediatric Surgery, Oslo University Hospital, 0450 Oslo, Norway
21 Department of Paediatric Surgery and Urology, Wrocław Medical University, Borowska 213, 50556 Wrocław, Poland
22 Department of Pediatric Surgery, Faculty of Medicine, Istanbul Medeniyet University, 34682 Istanbul, Turkey
23 Department of Neonatal Surgery, The Affiliated Children’s Hospital of Nanchang University, Nanchang 330006, China
24 Pediatric Clinic and Institute, University of Debrecen, 4032 Debrecen, Hungary
25 Department of Psychology, University of Bath, Building 10 West, Bath BA2 7AY, UK
26 Centre of Pediatric Surgery, Hannover Medical School, 30625 Hannover, Germany
27 Hochschule für Angewandte Wissenschaften Hamburg (HAW Hamburg), 20999 Hamburg, Germany

* Correspondence: michaela.m.blom@vgregion.se
‡ These authors contributed equally to this work.

### Appendix A.2. Author Contributions

This work reflects a group force. In that sense, each contribution is equally important to the study. M.D.B. was the principal investigator of this study, the international coordinator, and drafted the study design. All authors have read and agreed to the published version of the manuscript. The following co-authors were key in-country persons and national coordinators in their country: J.H.Q., J.D., N.D., S.E., A.S.G., A.R., I.S., K.B., K.E.M., B.Z., S.L. (Siqi Li), C.d.V., T.S., J.D.H.-P. and A.F. Draft conceptualization, M.D.B.; review and input to country-specific conceptualization, J.H.Q., J.D., N.D., S.E., A.S.G., A.R., I.S., K.B., K.E.M., B.Z., S.L. (Siqi Li), C.d.V., T.S., J.D.H.-P. and A.F.; methodology, M.D.B., S.W., J.D., J.H.Q., N.D., S.E., A.S.G., A.R., I.S., K.B., B.Z., K.E.M., S.L. (Siqi Li), A.F., C.d.V., T.S., J.D.P.H., G.S., AS., J.B., R.E., Z.A., R.S., D.P., Ç.U.D., M.S., F.G., D.Š.Š., T.L., S.G., S.Y., Y.Z., Y.G., S.L. (Shuangshuang Li), D.R.-A., O.K., MP., V.W., A.W., F.M.S., J.H., L.M.M., S.I., B.M.U. and D.S.; validation, M.D.B., J.H.Q., J.D., N.D., S.E., A.S.G., A.R., I.S., K.B., K.E.M., B.Z., S.L. (Siqi Li), C.d.V., T.S., J.D.H.-P. and A.F.; formal analysis, M.D.B.; J.H.Q., J.D., N.D., S.E., A.S.G., A.R., I.S., K.B., K.E.M., B.Z., S.L. (Siqi Li), C.d.V., T.S., J.D.H.-P. and A.F.; investigation, M.D.B., S.W., J.D., J.H.Q., N.D., S.E., A.S.G., A.R., I.S., K.B., B.Z., K.E.M., S.L. (Siqi Li), A.F., C.d.V., T.S., J.D.P.H., G.S., A.S., J.B., R.E., Z.A., R.S., D.P., Ç.U.D., M.S., F.G., D.Š.Š., T.L., S.G., S.Y., Y.Z., Y.G., S.L. (Shuangshuang Li), D.R.-A., O.K., M.P., V.W., A.W., F.M.S., J.H., L.M.M., S.I., B.M.U. and D.S.; supervision, J.H.Q., J.D., N.D., S.E., A.S.G., A.R., I.S., K.B., K.E.M., B.Z., S.L. (Siqi Li), C.d.V., T.S., J.D.H.-P. and A.F.; writing—original draft preparation, M.D.B.; writing—review and editing, M.D.B., S.W., J.D., J.H.Q., N.D., S.E., A.S.G., A.R., I.S., K.B., B.Z., K.E.M., S.L. (Siqi Li), A.F., C.d.V., T.S., J.D.P.H., G.S., A.S., J.B., R.E., Z.A., R.S., D.P., Ç.U.D., M.S., F.G., D.Š.Š., T.L., S.G., S.Y., Y.Z., Y.G., S.L. (Shuangshuang Li), D.R.-A., O.K., M.P., V.W., A.W., F.M.S., J.H., L.M.M., S.I., B.M.U. and D.S. All authors have read and agreed to the published version of the manuscript.
