# Supplementary material for: An International Collaborative Initiative to Establish a Quality-of-Life Questionnaire for Children and Adolescents with Repair of Esophageal Atresia in 14 Countries
Source: Children (Basel). 2024 Feb 26;11(3):286. doi: 10.3390/children11030286 (PMC10968779; doi:10.3390/children11030286)
Supplement: Supplementary file 1 [file children-11-00286-s001.zip › Supplementary File S2.pdf]

Supplementary File S2. Description of the data collection method used in the cognitive debriefing interviews of the EA-QOL questionnaire for children aged 8-18 listed in order of the earliest study year and country

Country	Study year	Recruitment source	Number of interviewers	Profession of interviewer	Care and treatment relationship	Location of the interviews
**Sweden-Germany**	2016	Clinical center	3	Pediatric Nurse, PhD student, Psychologist, PhD student	No	Clinical center/Hospital
**Turkey**	2019	Clinical center	2	Pediatric Surgeons	Yes	Clinical center/Hospital University
**Spain**	2019	Clinical center	1	Pediatric Surgeon	No	Clinical center/Hospital
**United Kingdom**	2020	Patient support group, TOFS	1	Pediatric Surgeon	No	Online/Virtual interviews
**USA**	2020	Clinical center	1	Quality improvement consultant	No	Clinical center/Hospital
**Croatia**	2020	Clinical center	3	Medical Doctor Professor of pedagogy Pediatric surgeon	No	Clinical center/Hospital
**Hungary**	2020	Clinical center, Patient support group	2	Pediatric gastroenterologist PhD student, a clinical laboratory scientist	No	University
**Poland**	2021	Clinical center	4	Pediatric Nurse, Geneticist, Pediatric Surgeons	Yes	Clinical center/Hospital
**China**	2021	Clinical center	1	Master degree candidate in medicine	No	Online/Virtual interviews
**South Africa**	2021	Clinical center	2	Registrar in paediatric surgery and rotating Intern	No	Clinical center/Hospital
**Norway**	2021-2022	Clinical center, Patient support group	3	Medical doctors, clinical nutritionist	Yes[1]/No	Clinical center/Hospital, Telephone (three interviews)
**France**	2021-2022	Clinical center	1	Psychologist	Yes	Clinical center/Hospital
**Mexico**	2022	Clinical center	2	Pediatric Surgeons	Yes	Clinical center/Hospital

[1]The researcher with the main responsibility of the cognitive debriefing interviews and conducted most of them did not have a care and treatment relationship with the children
