# Supplementary material for: An International Collaborative Initiative to Establish a Quality-of-Life Questionnaire for Children and Adolescents with Repair of Esophageal Atresia in 14 Countries
Source: Children (Basel). 2024 Feb 26;11(3):286. doi: 10.3390/children11030286 (PMC10968779; doi:10.3390/children11030286)
Supplement: Supplementary file 1 [file children-11-00286-s001.zip › Supplementary File S3.pdf]

Supplementary File S3

Supplementary File 3a-b describes the number and percentage of children with esophageal atresia aged 8-18 and their parent-proxies who rated the items of the EA-QOL questionnaire as easy to understand. The results from the cognitive debriefing of the EA-QOL questionnaire for children with esophageal atresia aged 8-18 are presented for Sweden-Germany[33] as a reference from the primary item evaluation and for Europe (Croatia, France, Hungary, Norway, Poland, Spain, Turkey, United Kingdom, Africa (South Africa), Asia (China[45]), Central America (Mexico) and North-America (USA) as the new item evaluation.

Supplementary file 3a. Number and percentage of children with esophageal atresia aged 8-18 who rated the item of the EA-QOL questionnaires as easy to understand															
			**EUROPE**									**AFRICA**	**ASIA**	**CENTRAL-AMERICA**	**NORTH-AMERICA**	**CROSS-CULTURAL**
			**Sweden-Germany (n=15)**	**Croatia (n=9)**	**France (n=4)**	**Hungary (n=6)**	**Norway(n=8)**	**Poland (n=9)**	**Spain (n=9)**	**Turkey (n=5)**	**United Kingdom (n=10)**	**South Africa (n=6)**	**China (n=6)**	**Mexico (n=4)**	**USA(n=6)**	**Cross-cultural (n=82)[c]**
EATING	1.	I feel distressed that food gets stuck in my throat when I eat	14(93.3)	9(100)	4(100)	6(100)	7(87.5)	9(100)	9(100)	5(100)	10(100)	6(100)	6(100)	4(100)	6(100)	81(98.8)
	2.	My health condition restricts me from eating certain foods'	14(93.3)	8(88.9)	4(100)	6(100)	8(100)	9(100)	9(100)	5(100)	9(90)	6(100)	6(100)	3(75)	6(100)	79(96.3)
	3.	It hurts when I eat because of my health condition (e.g. when food sticks, heartburn, tummy ache)	15(100)	9(100)	4(100)	6(100)	8(100)	9(100)	9(100)	4(80)	10(100)	6(100)	6(100)	4(100)	6(100)	81(98.8)
	4.	I have to remind myself to drink liquids when I eat	14(93.3)	9(100)	4(100)	6(100)	8(100)	9(100)	9(100)	5(100)	9(90)	6(100)	6(100)	4(100)	5(83.3)	80(97.6)
	5.	I am afraid when I choke while eating	15(100)	9(100)	4(100)	6(100)	8(100)	9(100)	9(100)	4(80)	9(90)	5(83.3)	6(100)	4(100)	6(100)	79(96.3)

	6.	I feel that my experiences of choking make it difficult for me to eat	14(93.3)	9(100)	4(100)	6(100)	7(87.5)	9(100)	9(100)	4(80)	9(90)	6(100)	6(100)	4(100)	6(100)	79(96.3)
	7.	I can eat at the same speed/pace as other children my age	15(100)	9(100)	4(100)	6(100)	7(87.5)	9(100)	8(88.9)	5(100)	10(100)	6(100)	6(100)	4(100)	6(100)	80(97.6)
	8.	It bothers me if I vomit after I eat	13(86.7)	8(88.9)	4(100)	6(100)	8(100)	9(100)	8(88.9)	4(80)	10(100)	6(100)	6(100)	4(100)	6(100)	79(96.3)
SOCIAL RELATIONSHIPS	9.	I feel like the only one who was born with esophageal atresia	14(100)[a]	8(88.9)	4(100)	6(100)	7(87.5)	9(100)	9(100)	4(80)	10(100)	6(100)	6(100)	4(100)	6(100)	79(96.3)
	10.	It is complicated to explain to others what esophageal atresia is	13(86.7)	8(88.9)	4(100)	5(83.3)	8(100)	9(100)	8(88.9)	5(100)	10(100)	6(100)	6(100)	4(100)	6(100)	79(96.3)
	11.	People call me names (perhaps because of your size, having an unusual cough, eating slowly, or	15(100)	8(88.9)	4(100)	6(100)	8(100)	9(100)	9(100)	4(80)	9(90)	6(100)	6(100)	4(100)	6(100)	79(96.3)

		because you have a surgical scar)														
	12.	I feel that other people are staring at me (e.g. when coughing, choking, dressing in the locker room)	13(86.7)	8(88.9)	4(100)	6(100)	8(100)	9(100)	9(100)	5(100)	10(100)	6(100)	6(100)	4(100)	6(100)	81(98.8)
	13.	I get tired of people asking about the scar/scars	14(93.3)	8(88.9)	4(100)	6(100)	8(100)	9(100)	9(100)	5(100)	10(100)	6(100)	6(100)	4(100)	6(100)	81(98.8)
	14.	Other people say unkind things about me	15(100)	8(88.9)	4(100)	6(100)	7(87.5)	9(100)	9(100)	5(100)	9(90)	6(100)	6(100)	4(100)	6(100)	79(96.3)
	15.	It feels awkward when other people ask me about esophageal atresia	14(93.3)	8(88.9)	4(100)	6(100)	7(87.5)	9(100)	9(100)	4(80)	10(100)	5(83.3)	6(100)	4(100)	6(100)	78(95.1)
BODY PERCEPTION	16.	I feel different because I have scars	15(100)	8(88.9)	4(100)	6(100)	8(100)	9(100)	9(100)	5(100)	10(100)	6(100)	6(100)	4(100)	5(83.3)	80(97.6)
	17.	I am careful about what I wear because of	14(93.3)	8(100)[a]	4(100)	6(100)	7(87.5)	9(100)	9(100)	5(100)	10(100)	5(83.3)	6(100)	4(100)	5(83.3)	78(96.3)[a]

		my scar /scars														
	18.	I feel awkward when my scar/scars are visible to other people (e.g. new people, boy or girlfriend, people in the changing room, or in the swimming pool)	13(86.7)	8(100)[a]	4(100)	6(100)	8(100)	9(100)	9(100)	5(100)	10(100)	6(100)	6(100)	4(100)	5(83.3)	80(98.8)
	19.	I am unhappy with the way I look because I have scars	15(100)	8(100)[a]	4(100)	6(100)	8(100)	9(100)	9(100)	5(100)	9(100)[a]	5(83.3)	6(100)	4(100)	5(83.3)	78(97.5)[b]
	20.	It bothers me that I am smaller than children my age	13(86.7)	7(87.5)[a]	4(100)	6(100)	8(100)	9(100)	9(100)	5(100)	10(100)	6(100)	6(100)	4(100)	5(83.3)	80(98.8)[a]
HEALTH & WELL-BEING	21.	I am bothered by breathing difficulties if I exercise and play	15(100)	8(100)[a]	4(100)	6(100)	8(100)	9(100)	9(100)	5(100)	9(90)	6(100)	6(100)	4(100)	6(100)	80(98.8)[a]
	22.	I have trouble falling or	14(93.3)	8(100)[a]	4(100)	6(100)	8(100)	9(100)	9(100)	5(100)	10(100)	5(83.3)	6(100)	4(100)	6(100)	80(98.8)[a]

		staying asleep at night because of my health condition. (e.g. acid reflux, heartburn, or respiratory problems)													
	23.	I am worried about my future because of esophageal atresia (e.g. school, friends, boy or girlfriend, work)	13(86.7)	8(100)[a]	4(100)	6(100)	7(87.5)	9(100)	9(100)	5(100)	10(100)	6(100)	6(100)	4(100)	6(100)	80(98.8)[a]
	24.	Esophageal atresia makes me sad	13(86.7)	8(100)[a]	4(100)	6(100)	8(100)	9(100)	9(100)	5(100)	10(100)	6(100)	6(100)	4(100)	6(100)	81(100)[a]

[a] 1 missing value
[b] 2 missing values
[c] excluding data from Sweden and Germany

Additional file 3b. Number and percentage of parents of children with esophageal atresia aged 8-18 who rated the item of the EA-QOL questionnaires as easy to understand															
			EUROPE									AFRICA	ASIA	CENTRAL-AMERICA	NORTH-AMERICA	CROSS-CULTURAL
			**Sweden-Germany (n=13)**	**Croatia (n=9)**	**France (n=5)**	**Hungary (n=6)**	**Norway (n=8)**	**Poland (n=9)**	**Spain (n=9)**	**Turkey (n=7)**	**United Kingdom (n=10)**	**South Africa (n=6)**	**China (n=6)**	**Mexico (n=5)**	**USA(n=6)**	**Cross-cultural (n=86)[c]**
EATING	1.	My child feels distressed that food gets stuck in their throat when they eat	13(100)	8(88.9)	5(100)	6(100)	6(75.0)	9(100)	9(100)	7(100)	8(80)	6(100)	6(100)	5(100)	6(100)	81(94.2)
	2.	My child's health condition restricts them from eating certain foods	12(92.3)	9(100)	5(100)	6(100)	7(87.5)	9(100)	9(100)	7(100)	8(80)	6(100)	6(100)	5(100)	6(100)	83(96.5)
	3.	My child's health condition causes pain when they eat (e.g. when food sticks, heartburn, tummy ache)	12(92.3)	9(100)	5(100)	6(100)	8(100)	9(100)	9(100)	7(100)	8(80)	6(100)	6(100)	5(100)	6(100)	84(97.4)
	4.	My child needs to remind themselves to drink liquids when they eat	11(91.7)	9(100)	5(100)	6(100)	7(87.5)	9(100)	8(88.9)	7(100)	6(60)	6(100)	6(100)	5(100)	6(100)	80(93.0)
	5.	My child is afraid when they choke while eating	13(100)	9(100)	5(100)	6(100)	6(75.0)	9(100)	9(100)	7(100)	7(70)	6(100)	6(100)	5(100)	6(100)	81(94.2)
	6.	My child's choking experiences make it difficult for them to eat	13(100)	9(100)	5(100)	6(100)	7(87.5)	9(100)	9(100)	7(100)	6(60)	6(100)	6(100)	5(100)	6(100)	81(94.2)

	7.	My child can eat at the same speed/pace as other children their age	13(100)	9(100)	5(100)	6(100)	8(100)	9(100)	9(100)	7(100)	7(70)	6(100)	6(100)	5(100)	6(100)	83(96.5)
	8.	It bothers my child if they vomit after eating	13(100)	8(88.9)	5(100)	6(100)	7(87.5)	9(100)	7(77.8)	6(85.7)	3(30)	6(100)	6(100)	5(100)	6(100)	74(86.0)
SOCIAL RELATIONSHIPS	9.	My child feels like to only one to be born with esophageal atresia	13(100)	9(100)	5(100)	5(83.3)	8(100)	9(100)	9(100)	7(100)	10(100)	6(100)	6(100)	5(100)	6(100)	85(98.8)
	10.	My child finds it complicated to explain to others what esophageal atresia is	13(100)	9(100)	5(100)	6(100)	6(85.7)[a]	9(100)	8(88.9)	7(100)	10(100)	5(83.3)	6(100)	5(100)	6(100)	82(96.5)[a]
	11.	My child is called names (e.g. because of their size, unusual cough, slow eating or because of their scar/scars)	13(100)	9(100)	5(100)	6(100)	7(87.5)	9(100)	9(100)	7(100)	10(100)	6(100)	6(100)	5(100)	6(100)	85(98.8)
	12.	My child feel that other stare at them (e.g. when coughing, choking, dressing in the locker room)	13(100)	9(100)	5(100)	6(100)	8(100)	9(100)	9(100)	7(100)	10(100)	6(100)	6(100)	5(100)	6(100)	86(100)
	13.	My child gets tired of people asking about their scar/scars	12(92.3)	9(100)	5(100)	6(100)	8(100)	9(100)	9(100)	7(100)	10(100)	6(100)	6(100)	5(100)	6(100)	86(100)

	14.	Other people say unkind things about my child	12(92.3)	9(100)	5(100)	6(100)	6(75.0)	9(100)	9(100)	7(100)	7(70)	6(100)	6(100)	5(100)	6(100)	81(94.2)
	15.	My child feels awkward when other people ask them about esophageal atresia	13(100)	9(100)	5(100)	6(100)	6(75.0)	9(100)	9(100)	7(100)	10(100)	6(100)	6(100)	5(100)	6(100)	84(97.7)
BODY PERCEPTION	16.	My child feels different because they have a scar/scars	13(100)	8(88.9)	5(100)	6(100)	8(100)	9(100)	9(100)	7(100)	10(100)	6(100)	6(100)	5(100)	6(100)	85(98.8)
	17.	My child is careful about what they wear because of their scar/scars	13(100)	8(88.9)	5(100)	6(100)	8(100)	9(100)	9(100)	7(100)	10(100)	6(100)	6(100)	5(100)	6(100)	85(98.8)
	18.	My child feels awkward when their scar/scars are visible to other people	13(100)	8(88.9)	5(100)	6(100)	8(100)	9(100)	9(100)	7(100)	8(80)	6(100)	6(100)	5(100)	6(100)	83(96.5)
	19.	My child is unhappy with the way they look because of their scar/scars	13(100)	8(88.9)	5(100)	6(100)	7(87.5)	9(100)	9(100)	7(100)	7(77.8)[a]	6(100)	6(100)	5(100)	6(100)	81(95.3)[a]
	20.	My child is bothered that they are smaller than children their age	11(100)[b]	8(88.9)	5(100)	6(100)	8(100)	9(100)	9(100)	7(100)	10(100)	6(100)	6(100)	5(100)	6(100)	85(98.8)
HEALTH & WELL	21.	My child is bothered by breathing difficulties if	13(100)	8(88.9)	5(100)	6(100)	8(100)	9(100)	9(100)	7(100)	9(90)	6(100)	6(100)	5(100)	6(100)	84(97.7)

		they exercise and play													
	22.	My child has trouble falling or staying asleep at night because of their health condition(e.g. acid reflux, heartburn, or respiratory problems)	13(100)	7(77.8)	5(100)	6(100)	8(100)	9(100)	9(100)	7(100)	8(80)	6(100)	6(100)	5(100)	6(100)	82(95.3)
	23.	My child worries about their future because of esophageal atresia (e.g. school, friends, boy or girlfriend, work)	12(100)[a]	7(77.8)	5(100)	6(100)	6(75.0)	9(100)	9(100)	7(100)	10(100)	6(100)	6(100)	5(100)	6(100)	82(95.3)
	24.	My child feels sad because of esophageal atresia	12(100)[a]	7(77.8)	5(100)	6(100)	7(87.5)	9(100)	9(100)	7(100)	9(90)	6(100)	6(100)	5(100)	6(100	82(95.3)

[a] 1 missing
[b] 2 missing
[c] excluding data from Sweden and Germany

Supplementary file 3c-d.

Supplementary file 3c-d describes the number and percentage of children with esophageal atresia aged 8-18 and their parent-proxies who rated the items of the EA-QOL questionnaire as sensitive to answer. The results from the cognitive debriefing of the EA-QOL questionnaire for children with esophageal atresia aged 8-18 are presented for Sweden-Germany[1] as a reference from the primary item evaluation and for Europe (Croatia, France, Hungary, Norway, Poland, Spain, Turkey, United Kingdom, Africa (South Africa), Asia (China[2]), Central America (Mexico) and North-America (USA) as the new item evaluation.

Supplementary file 3c. Number and percentage of children with esophageal atresia aged 8-18 who rated the item of the EA-QOL questionnaire as sensitive to answer

			EUROPE									AFRICA	ASIA	CENTRAL-AMERICA	NORTH-AMERICA	CROSS-CULTURAL
			**Sweden-Germany (n=15)**	**Croatia (n=9)**	**France (n=4)**	**Hungary (n=6)**	**Norway(n=8)**	**Poland (n=9)**	**Spain (n=10)**	**Turkey (n=5)**	**United Kingdom (n=10)**	**South Africa (n=6)**	**China (n=6)**	**Mexico (n=4)**	**USA(n=6)**	**Cross-cultural(n=82)[c]**
**EATING**	1.	I feel distressed that food gets stuck in my throat when I eat	0	0	0	0	1(12.5)	0	0	1(20.0)	0	1(16.7)	0	0	0	3(3.7)
	2.	My health condition restricts me from eating certain foods'	1(6.7)	0	0	0	0	0	0	0	0	1(16.7)	0	0	0	1(1.2)
	3.	It hurts when I eat because of my health condition (e.g. when food sticks, heartburn, tummy ache)	0	0	0	0	0	0	0	0	0	0	0	0	1(16.7)	1(1.2)
	4.	I have to remind myself to drink liquids when I eat	0	0	0	0	0	0	0	1(20.0)	0	1(16.7)	0	0	0	2(2.4)
	5.	I am afraid when I choke while eating	0	0	0	0	0	0	0	1(20.0)	0	1(16.7)	0	0	0	2(2.4)
	6.	I feel that my experiences of choking make it difficult for me to eat	2(13.3)	0	0	0	0	0	0	0	0	0	0	0	1(16.7)	1(1.2)
	7.	I can eat at the same speed/pace as other children my age	0	0	0	0	0	0	0	0	0	1(16.7)	0	0	0	1(1.2)

	8.	It bothers me if I vomit after I eat	0	0	0	0	0	0	0	1(20.0)	0	0	0	0	0	1(1.2)
SOCIAL RELATIONSHIPS	9.	I feel like the only one who was born with esophageal atresia	1(7.1)[a]	0	0	0	0	0	0	0	2(20)	2(33.3)	0	0	1(16.7)	5(6.1)
	10.	It is complicated to explain to others what esophageal atresia is	1(6.7)	0	0	0	0	0	0	0	0	1(16.7)	0	0	0	1(1.2)
	11.	People call me names (perhaps because of your size, having an unusual cough, eating slowly, or because you have a surgical scar)	1(6.7)	0	0	0	0	0	0	2(40.0)	0	2(33.3)	0	0	1(16.7)	5(6.1)
	12.	I feel that other people are staring at me (e.g. when coughing, choking, dressing in the locker room)	0	0	0	0	0	0	0	1(20.0)	2(20)	2(33.3)	0	0	1(16.7)	6(7.3)
	13.	I get tired of people asking about the scar/scars	1(6.7)	0	0	0	0	0	0	0	0	2(33.3)	0	0	1(16.7)	3(3.7)
	14.	Other people say unkind things about me	0	0	0	0	0	0	0	0	0[a]	1(16.7)	0	0	1(16.7)	2(2.5)[a]
	15.	It feels awkward when other people ask me about	1(6.7)	0	0	0	0	0	0	1(20.0)	0	0	0	0	1(16.7)	2(2.4)

		esophageal atresia														
BODY PERCEPTION	16.	I feel different because I have scars	1(6.7)	0	0	0	0	0	0	0	0	2(33.3)	0	0	2(33.3)	4(4.9)
	17.	I am careful about what I wear because of my scar /scars	0	0[a]	0	0	0	0	0	1(20.0)	0	2(33.3)	0	0	2(33.3)	5(6.2)[a]
	18.	I feel awkward when my scar/scars are visible to other people (e.g. new people, boy or girlfriend, people in the changing room, or in the swimming pool)	1(6.7)	0[a]	0	0	0	0	0	1(20.0)	0	1(16.7)	0	0	2(33.3)	4(4.9)[a]
	19.	I am unhappy with the way I look because I have scars	2(13.3)	0[a]	0	0	1(12.5)	0	0	1(20.0)	2(22.2)[a]	1(16.7)	0	0	2(33.3)	7(8.8)[b]
	20.	It bothers me that I am smaller than children my age	1(6.7)	0[a]	0	0	0	0	0	1(20.0)	0	1(16.7)	0	0	2(33.3)	4(4.9)[a]
HEALTH & WELL-BEING	21.	I am bothered by breathing difficulties if I exercise and play	1(6.7)	0[a]	0	0	0	0	0	0	0	1(16.7)	0	0	2(33.3)	3(3.7)[a]
	22.	I have trouble falling or staying asleep at night because of my health condition. (e.g. acid reflux, heartburn, or	0	0[a]	0	0	0	0	0	0	0	0	0	0	1(16.7)	1(1.2)[a]

		respiratory problems)														
	23.	I am worried about my future because of esophageal atresia (e.g. school, friends, boy or girlfriend, work)	0	0[a]	0	0	0	0	0	0	0	1(20)[a]	0	0	2(33.3)	3(3.8)[b]
	24.	Esophageal atresia makes me sad	1(6.7)	0[a]	1(25.0)	0	0	0	0	0	4(40.0)	2(33.3)	0	0	1(16.7)	8(9.9)[a]

[a] 1 missing value
[b] 2 missing values
[c] excluding data from Sweden and Germany

Supplementary file 3d. Number and percentage of parents of children with esophageal atresia aged 8-18 who rated the item of the EA-QOL questionnaire as sensitive to answer																
			EUROPE									AFRICA	ASIA	CENTRAL-AMERICA	NORTH-AMERICA	CROSS-CULTURAL
			**Sweden-Germany (n=13)**	**Croatia (n=9)**	**France (n=5)**	**Hungary (n=6)**	**Norway (n=8)**	**Poland (n=9)**	**Spain (n=9)**	**Turkey (n=7)**	**United Kingdom (n=10)**	**South Africa (n=6)**	**China (n=6)**	**Mexico (n=5)**	**USA(n=6)**	**Cross-cultural (n=86)[c]**
EATING	1.	My child feels distressed that food gets stuck in their throat when they eat	0[a]	0	0	0	0	0	0	0	0	0	0	0	0	0
	2.	My child's health condition restricts them from eating certain foods	0[a]	0	0	0	1(12.5)	0	0	0	0	0	0	0	0	1(1.2)
	3.	My child's health condition causes pain when they eat (e.g. when food sticks, heartburn, tummy ache)	0[a]	0	0	0	0	0	0	0	0	1(16.7)	0	0	0	1(1.2)
	4.	My child needs to remind themselves to drink liquids when they eat	0[b]	0	0	0	1(12.5)	0	0	0	0	1(16.7)	0	0	0	2(2.3)
	5.	My child is afraid when they choke while eating	0[a]	0	0	0	0	0	0	0	1(10.0)	1(16.7)	0	0	0	2(2.3)
	6.	My child's choking experiences make it difficult for them to eat	0[a]	0	0	0	0	0	0	0	0	1(16.7)	0	0	0	1(1.2)
	7.	My child can eat at the same speed/pace as	1(8.3)[a]	0	0	0	0	0	0	0	1(10.0)	1(16.7)	0	0	0	2(2.3)

		other children their age														
	8.	It bothers my child if they vomit after eating	0[s]	0	0	0	1(12.5)	0	0	1(14.3)	0	1(16.7)	0	0	0	3(3.5)
SOCIAL RELATIONSHIPS	9.	My child feels like to only one to be born with esophageal atresia	1(8.3)[a]	0	0	1(16.7)	1(12.5)	0	0	0	2(20.0)	2(33.3)	0	0	0	6(7.0)
	10.	My child finds it complicated to explain to others what esophageal atresia is	0[a]	0	0	0	0	0	0	0	0	1(16.7)	0	0	0	1(1.2)
	11.	My child is called names (e.g. because of their size, unusual cough, slow eating or because of their scar/scars)	1(8.3)[a]	0	0	0	1(12.5)	0	0	0	5(50.0)	2(33.3)	0	0	0	8(9.3)
	12.	My child feel that other stare at them (e.g. when coughing, choking, dressing in the locker room)	0[a]	0	0	0	0	0	0	0	2(20.0)	0	0	0	0	2(2.3)
	13.	My child gets tired of people asking about their scar/scars	0[a]	0	0	0	0	0	0	0	0	1(16.7)	0	0	0[a]	1(1.2)[a]
	14.	Other people say unkind things about my child	1(8.3)[a]	0	0	0	3(37.5)	0	0	0	5(50.0)	0	0	0	0	8(9.3)
	15.	My child feels awkward when other people ask them about	0[b]	0	0	0	1(14.3)[a]	0	0	0	0	1(16.7)	0	0	1(16.7)	3(3.5)[a]

		esophageal atresia														
BODY PERCEPTION	16.	My child feels different because they have a scar/scars	0	0	0	0	1(12.5)	0	0	0	2(20.0)	1(16.7)	0	0	0	4(4.7)
	17.	My child is careful about what they wear because of their scar/scars	0	0	0	0	1(12.5)	0	0	0	0	1(16.7)	0	0	0	2(2.3)
	18.	My child feels awkward when their scar/scars are visible to other people	0	0	0	0	3(37.5)	0	0	0	3(30.0)	0	0	0	0	6(7.0)
	19.	My child is unhappy with the way they look because of their scar/scars	0	0	0	0	2(25.0)	0	0	0	6(60.0)	2(33.3)	0	0	0	10(11.6)
	20.	My child is bothered that they are smaller than children their age	2(16.7)[a]	0	0	0	2(25.0)	0	0	0	0	0	0	0	0	2(2.3)
HEALTH & WELL-BEING	21.	My child is bothered by breathing difficulties if they exercise and play	1(8.3)[a]	0	0	0	0	0	0	0	0	2(33.3)	0	0	0	2(2.3)
	22..	My child has trouble falling or staying asleep at night because of their health condition(e.g. acid reflux, heartburn, or	0	0	0	0	0	0	0	0	1(10)	1(16.7)	0	0	0	2(2.3)

		respiratory problems)														
	23.	My child worries about their future because of esophageal atresia (e.g. school, friends, boy or girlfriend, work)	0[a]	0	0	0	1(12.5)	0	0	0	2(20.0)	1(16.7)	0	0	0	4(4.67
	24.	My child feels sad because of esophageal atresia	1(8.3)[a]	0	0	0	0	0	0	0	2(20.0)	1(16.7)	0	0	0	3(3.5)

[a] 1 missing value
[b] 2 missing values
[c] excluding data from Sweden and Germany

References as cited in the main manuscript

[33] Dellenmark-Blom M, Abrahamsson K, Quitmann JH, Sommer R, Witt S, Dingemann J, et al. Development and pilot-testing of a condition-specific instrument to assess the quality-of-life in children and adolescents born with esophageal atresia. Diseases of the Esophagus 2017;30(7):1-9

[45] Li S, Dellenmark-Blom M, Zhao Y, Gu Y, Li S, Yang S, Quitmann JH, Huang J. The Chinese Mandarin Version of the Esophageal-Atresia-Quality-of-Life Questionnaires for Children and Adolescents: Evaluation of Linguistic and Content Validity. Int J Environ Res Public Health. 13;19(22):14923. doi: 10.3390/ijerph192214923.
