# Supplementary material for: An International Collaborative Initiative to Establish a Quality-of-Life Questionnaire for Children and Adolescents with Repair of Esophageal Atresia in 14 Countries
Source: Children (Basel). 2024 Feb 26;11(3):286. doi: 10.3390/children11030286 (PMC10968779; doi:10.3390/children11030286)
Supplement: Supplementary file 1 [file children-11-00286-s001.zip › Supplementary File S4.pdf]

Supplementary File 4

Categorization of children's (4a) and parents' (4b) comments of the understanding and perceived difficulties of items in the EA-QOL questionnaire for children with esophageal atresia aged 8-18. The categorization is based on their open comments in cognitive debriefing interviews as documented in field notes by the interviewer. The number of parents who confirmed understanding of the item or described a type of item difficulty is presented. While 82 children and 86 parents of children with EA aged 8-18 participated in the cognitive debriefing interviews, between 2-10 children and 0-13 parents commented on the items in own words.

Supplementary File 4a. Children's understanding and perceived difficulties of items in the EA-QOL questionnaire for children with esophageal atresia aged 8-18

		Recognition and understanding of the situation in context of EA		Unclear wording/Ambiguity		Difficult to answer if no current experience of symptoms/the situation		Difficult to answer due to young child age		Emotive question/strong expression		Suggested improvement
		n_{tot}	Country (number of parents making the statement)	n_{tot}	(number of parents making the statement)	n_{tot}	(number of parents making the statement)	n_{tot}	(number of parents making the statement)	n_{tot}	(number of parents making the statement)	(number of parents making the statement)
1.	I feel distressed that food gets stuck in my throat when I eat	7	China (n=6)<br>South Africa (n=1)	8	China (n=5)<br>United Kingdom (n=1)<br>Croatia (n=1)<br>Norway (n=1)	0		0		0		China (n=5), incorporated
2.	My health condition restricts me from eating certain foods'	9	China (n=6)<br>United Kingdom (n=1)<br>Spain (n=1)<br>South Africa (n=1)	2	Croatia (n=1)<br>Mexico (n=1)	0		0		0		United Kingdom (n=1), incorporated<br>Mexico (n=1), incorporated
3.	It hurts when I eat because of my health condition (e.g. when food sticks, heartburn, tummy ache)	10	China (n=6)<br>United Kingdom (n=4)	0		0		0		0		
4.	I have to remind myself to drink liquids when I eat	7	China (n=6)<br>South Africa (n=1)	2	United Kingdom (n=2)	0		0		0		
5.	I am afraid when I choke while eating	9	China (n=6)<br>Hungary (n=2)<br>South Africa (n=1)	9	China (n=6)<br>United Kingdom (n=3)	0		0		0		China (n=6), incorporated
6.	I feel that my experiences of choking make it difficult for me to eat	6	China (n=6)	9	China (n=6)<br>Turkey (n=1)<br>United Kingdom (n=1)<br>Norway (n=1)	0		0		0		China (n=6), incorporated
7.	I can eat at the same speed/pace as other children my age	7	China (n=6)<br>South Africa (n=1)	1	Spain (n=1)	0		0		0		
8.	It bothers me if I vomit after I eat	12	China (n=6)<br>United Kingdom (n=4)<br>Turkey (n=1)<br>South Africa (n=1)	8	United Kingdom (n=5)<br>Turkey (n=1)<br>Spain (n=1)<br>Norway(n=1)	1	Croatia (n=1)	0		0		
9.	I feel like the only one who was born with esophageal atresia	7	China (n=6)<br>South Africa (n=1)	0		1	Hungary (n=1)	1	Croatia (n=1)	2	United Kingdom (n=2)	
10.	It is complicated to explain to others what esophageal atresia is	4	Spain (n=3)<br>South Africa (n=1)	0		5	China (n=3)<br>Croatia (n=1)<br>United Kingdom (n=1)	1	Croatia (n=1)			

11.	People call me names (perhaps because of your size, having an unusual cough, eating slowly, or because you have a surgical scar)	9	China (n=6)<br>United Kingdom (n=1)<br>South Africa (n=1)<br>Mexico (n=1)	2	United Kingdom (n=1)<br>Norway (n=1)	0		1	Croatia (n=1)	0		United Kingdom (n=2)
12.	I feel that other people are staring at me (e.g.when coughing, choking, dressing in the locker room)	2	China (n=1)<br>South Africa (n=1)	0		0		1	Croatia (n=1)	1	United Kingdom (n=1)	
13.	I get tired of people asking about the scar/scars	4	China (n=3)<br>South Africa (n=1)	1	Croatia (n=1)	0		0		0		
14.	Other people say unkind things about me	9	China (n=6)<br>Spain (n=2)<br>South Africa (n=1)	4	United Kingdom (n=2)<br>Croatia (n=1)<br>Norway (n=1)			0		0		
15.	It feels awkward when other people ask me about esophageal atresia	2	China (n=2)	2	South Africa (n=1)<br>United Kingdom (n=1)	5	Croatia (n=1)<br>Hungary (n=1)<br>United Kingdom (n=1)<br>China (n=1)<br>South Africa (n=1)	0		0		
16.	I feel different because I have scars	8	China (n=6)<br>Hungary (n=1)<br>France (n=1)	1	Croatia (n=1)	0		0		0		
17.	I am careful about what I wear because of my scar /scars	9	China (n=6)<br>France (n=1)<br>South Africa (n=2)	1	Croatia (n=1)	0		0		0		United Kingdom (n=1)
18.	I feel awkward when my scar/scars are visible to other people (e.g. new people, boy or girlfriend, people in the changing room, or in the swimming pool)	8	China (n=5)<br>United Kingdom (n=1)<br>France (n=1)<br>South Africa (n=1)	2	Croatia (n=1)<br>United Kingdom (n=1)	1	China (n=1)	0		0		
19.	I am unhappy with the way I look because I have scars	8	China (n=6)<br>France (n=1)<br>South Africa (n=1)	1	Croatia (n=1)	0		0		1	United Kingdom (n=1)	United Kingdom (n=1), incorporated
20.	It bothers me that I am smaller than children my age	7	China (n=6)<br>South Africa (n=1)	3	Croatia (n=2)<br>United Kingdom (n=1)	1	United Kingdom (n=1)	0		0		United Kingdom (n=1)
21.	I am bothered by breathing difficulties if I exercise and play	9	China (n=6)<br>Spain (n=2)<br>South Africa (n=1)	3	Croatia (n=1)<br>United Kingdom (n=2)	0		0		0		United Kingdom (n=2)
22.	I have trouble falling or staying asleep at night because of my health condition. (e.g. acid reflux, heartburn, or respiratory problems)	8	China (n=6)<br>Hungary (n=1)<br>Spain (n=1)	1	Croatia (n=1)	0		0		0		

23.	I am worried about my future because of esophageal atresia (e.g. school, friends, boy or girlfriend, work)	8	China (n=6)<br>United Kingdom (n=1)<br>Spain (n=1)	1	Croatia (n=1)	0		0		2	France (n=1)<br>United Kingdom (n=1)	
24.	Esophageal atresia makes me sad	10	United Kingdom (n=2)<br>Spain (n=1)<br>(China n=6)<br>South Africa (n=1)	1	Croatia (n=1)	0		0		3	United Kingdom (n=3)	

	Supplementary File 4b. Categorization of parents' understanding and perceived difficulties of items in the EA-QOL questionnaire for children with esophageal atresia aged 8-18															
		**Recognition and understanding of the situation in context of EA**		**Unclear wording/Ambiguity**		**Difficult to answer if no current experience of symptoms/the situation**		**Bothers parents/impacts the family rather than the child**		**Emotive question/strong expression**		**Child too young**		**Parent unsure/ doesn't know**		**Suggested improvement**
		n_{tot}	(number of parents making the statement)	n_{tot}	(number of parents making the statement)	n_{tot}	(number of parents making the statement)	n_{tot}	(number of parents making the statement)	n_{tot}	(number of parents making the statement)	n_{tot}	(number of parents making the statement)	n_{tot}	(number of parents making the statement)	(number of parents making the statement)
1.	My child feels distressed that food gets stuck in their throat when they eat	7	China (n=5)<br>Hungary (n=1)<br>Croatia (n=1)	9	United Kingdom (n=2)<br>Norway (n=2)<br>China (n=5)	1	Croatia (n=1)	0		0		0		0		Croatia (n=1<br>United Kingdom (n=1)<br>Norway (n=1), incorporated
2.	My child's health condition restricts them from eating certain foods	5	China (n=5)	0		1	China (n=1)	0		0		0		0		United Kingdom (n=2)
3.	My child's health condition causes pain when they eat (e.g. when food sticks, heartburn, tummy ache)	5	China (n=4)<br>Hungary (n=1)	1	France (n=1)	0		0		0		0		2	China (n=1)<br>France (n=1)	United Kingdom (n=6)
4.	My child needs to remind themselves to drink liquids when they eat	6	China (n=6)	2	United Kingdom (n=1)<br>Spain (n=1)	0		0		0		0		0		United Kingdom (n=2)
5.	My child is afraid when they choke while eating	5	China (n=3)<br>Hungary (n=2)	13	China (n=6)<br>United Kingdom (n=6)<br>Norway (n=1)	0		0		0	United Kingdom (n=1)	0		0		

6.	My child's choking experiences make it difficult for them to eat	0		11	China (n=6)<br>United Kingdom (n=4)<br>Norway (n=1)	0		0		0		0		0		Norway (n=1), incorporated United Kingdom (n=2)
7.	My child can eat at the same speed/pace as other children their age	8	China (n=6)<br>United Kingdom (n=2)	0		1	Hungary (n=1)	0		0		0		0		United Kingdom (n=1)
8.	It bothers my child if they vomit after eating	3	China (n=2)<br>Spain (n=1)	8	United Kingdom (n=6)<br>Croatia (n=1)<br>Spain (n=1)	4	China (n=3)<br>Spain (n=1)	1	China (n=1)	0		0		0		United Kingdom (n=1), incorporated
9.	My child feels like to only one to be born with esophageal atresia	5	China (n=4)<br>Hungary (n=1)	0		0		0		1	United Kingdom (n=1)	0		3	China (n=2)<br>United Kingdom (n=1)	
10.	My child finds it complicated to explain to others what esophageal atresia is	1	Hungary (n=1)	0		0		0		1	United Kingdom (n=1)	0		1	United Kingdom (n=1)	United Kingdom (n=1)
11.	My child is called names (e.g. because of their size, unusual cough, slow eating or because of their scar/scars)	3	Hungary (n=1)<br>USA (n=1)<br>China (n=1)	0		5	China (n=5)	1	China (n=1)	2	United Kingdom (n=1)<br>Norway (n=1)	0		1	United Kingdom (n=1)	United Kingdom (n=1)
12.	My child feel that other stare at them (e.g. when coughing, choking, dressing in the locker room)	5	China (n=5)	1	United Kingdom (n=1)	1	China (n=1)	0		2	United Kingdom (n=1)<br>Norway (n=1)	0		1	United Kingdom (n=1)	United Kingdom (n=2)
13.	My child gets tired of people asking about their scar/scars	3	China (n=3)	1	United Kingdom (n=1)	3	China (n=3)	0		1	Norway (n=1)	0		1	United Kingdom (n=1)	United Kingdom (n=1)
14.	Other people say unkind things about my child	1	Turkey (n=1)	2	United Kingdom (n=1)<br>Norway (n=1)	0		0		4	United Kingdom (n=4)	0		0		United Kingdom (n=2), incorporated
15.	My child feels awkward when other people ask them about esophageal atresia	3	China (n=3)	2	United Kingdom (n=1)<br>Norway (n=1)	3	China (n=3)	0		1	Norway (n=1)	0		0		United Kingdom (n=1)
16.	My child feels different because they have a scar/scars	10	China (n=6)<br>Croatia (n=1)	2	Croatia (n=2)	0		0		0		0		0		

			United Kingdom (n=2) South Africa (n=1)													
17.	My child is careful about what they wear because of their scar/scars	7	China (n=6) South Africa (n=1)	2	Croatia (n=1) United Kingdom (n=1)	0		0		1	United Kingdom (n=1)	0		0		
18.	My child feels awkward when their scar/scars are visible to other people	7	China (n=6) South Africa (n=1)	2	Croatia (n=1) United Kingdom (n=1)	1	France (n=1)	0		0		0		0		United Kingdom (n=1)
19.	My child is unhappy with the way they look because of their scar/scars	6	China (n=5) South Arica (n=1)	2	Croatia (n=1) United Kingdom (n=1)	1	China (n=1)	0		5	United Kingdom (n=5)	0		0		United Kingdom (n=2)
20.	My child is bothered that they are smaller than children their age	5	China (n=5)	3	Croatia (n=2) United Kingdom (n=1)	3	Hungary (n=2) United Kingdom (n=1)	1	China (n=1)	0		0		0		United Kingdom (n=2)
21.	My child is bothered by breathing difficulties if they exercise and play	7	China (n=6) South Africa (n=1)	3	United Kingdom (n=3)	0		0		1	Norway (n=1)	1	Croatia (n=1)	1	Croatia (n=1)	United Kingdom (n=1)
22.	My child has trouble falling or staying asleep at night because of their health condition(e.g. acid reflux, heartburn, or respiratory problems)	6	China (n=6)	4	United Kingdom (n=3) Croatia (n=1)	0		0		0		1	Croatia (n=1)	1	Croatia (n=1)	United Kingdom (n=3), incorporated
23.	My child worries about their future because of esophageal atresia (e.g. school, friends, boy or girlfriend, work)	5	China (n=5)	1	France (n=1)	1	China (n=1)	1	United Kingdom (n=1)	0		1	Croatia (n=1)	0		
24.	My child feels sad because of esophageal atresia	6	China (n=6)	0		0		0		1	United Kingdom (n=1)	1	Croatia (n=1)	1	Croatia (n=1)	Croatia (n=1)
