# Supplementary material for: An International Collaborative Initiative to Establish a Quality-of-Life Questionnaire for Children and Adolescents with Repair of Esophageal Atresia in 14 Countries
Source: Children (Basel). 2024 Feb 26;11(3):286. doi: 10.3390/children11030286 (PMC10968779; doi:10.3390/children11030286)
Supplement: Supplementary file 1 [file children-11-00286-s001.zip › Supplementary File S5.pdf]

Supplementary File 5. Implications of cognitive debriefing, expert review and harmonization of the EA-QOL questionnaires for children aged 8-18 in individual countries

As shown in Table 5, in five countries (Croatia, France, Hungary, Poland, and Turkey), no changes in the wording of the translated items were judged needed. Most improvements of the translations were conducted of items included in the domain eating. Corresponding to the input from children and parents, three items in Chinese Mandarin and Norwegian were improved in wording.

The translation of the UK, US, and South African English translations of the EA-QOL questionnaire needed several adaptations. Following the cognitive debriefing results, discussion with native experts (BZ, JB, ND, SE, CdV), patient representative native in English (GS), and the instrument developer (MDB), it was decided to convert all 24 questions into statements and replace he/she with a gender-neutral term "they" in the UK and US English translation to improve item clarity. Moreover, ten items in the UK-US English translation were improved in wording to increase clarity (items 1-2, 4-8, 14, 22) or to lessen the emotive expression (item 19). In the South African English version of the EA-QOL questionnaire, seven items were modified in wording in agreement with changes in the UK and US. In order to increase the applicability to the SA populations, three items were also simplified in the wording (e.g., "awkward" changed to "strange/uncomfortable"), and six items were added examples such as "restrict you from eating certain food (e.g., cannot eat everything?)". The native experts and patient representative of South Africa (CdV) also proposed that it is necessary to keep an interview-based approach with questions, not statements, which was agree upon with the EAT representative (GS) and instrument developer (MDB). Moreover, the wording using he/she was remained in the questions, as "they" was rarely applied in South African community.

After cognitive debriefing, the three languages who used the English version for translation into their languages, compared their version with English version again. This did not result in any need for changes in the Hungarian or the Chinese Mandarin version of the EA-QOL questionnaire. The European Spanish and Mexican Spanish version were compared with each other, the final US English version and the Swedish-German version, with discussions between the native experts (AGS, JDHP), a bilingual Spanish-English speaker (BZ) and the instrument developer (MDB). Given the cognitive debriefing results, expert review and harmonization all but changes in item wording, but one (item 1), were made because of the need for harmonization, thereby an equivalent European Spanish-Mexican Spanish version was created.
