# Supplementary material for: An International Collaborative Initiative to Establish a Quality-of-Life Questionnaire for Children and Adolescents with Repair of Esophageal Atresia in 14 Countries
Source: Children (Basel). 2024 Feb 26;11(3):286. doi: 10.3390/children11030286 (PMC10968779; doi:10.3390/children11030286)
Supplement: Supplementary file 1 [file children-11-00286-s001.zip › Supplemetary file S1.pdf]

Supplementary file S1. The target languages, year of establishment, key in-country consultants, professionals involved in the forward-backward translation, and result of the back-translation review

The UK and US English translations of the EA-QOL questionnaire were completed already in 2015, and in conjunction with the establishment of the study protocol, it was again professionally reviewed by a native English speaker for US and UK English in 2017. The remaining nine new translations were conducted between 2018-2022.

In each country, a key in-country consultant managed the process in the target country and was the primary contact person with the instrument developer (MDB). To achieve the forward-backward translation, the research team in four countries used translators in a healthcare profession; in three countries, the team recruited translators from a professional agency, whereas both options were used in four countries. Following two independent forward-translations, reconciliation and a back-translation, the semantical agreement/disagreements between the back-translation and source version of the EA-QOL questionnaire were recorded by the instrument developer (MDB) and the key in-country consultant using a standardized excel-file for documentation. In the three countries (China, Hungary, Spain) where the research team was permitted to use the English version of the EA-QOL questionnaire for translation into the new language, a procedure which has previously been described in detail for the Chinese Mandarin language [45]. If the English version was treated as source version, the back translation review was additionally conducted by a native English speaker fluent in Swedish who was recruited for this purpose only. If the back-translation review indicated a need to revise the new translation, it was discussed between the native experts and instrument developer (MDB) until a consensus on a good translation was reached.

To promote conceptual equivalence across all translations, the instrument developer and/or the key in-country consultant used the study protocol with description of the aim of the items, item key words and quotes from families of children with EA who participated in the Swedish focus groups during the process.

Supplemental File S1. The target languages, year of establishment, key in-country consultants, actors involved in the forward-backward translation, and result of the back-translation review

**New languages**	**Source language**	**Key-in country consultant**	**Forward-backward translation**			**Back-translation review**	
**Target language**	Language used for translation to the target language	Main contact person managing the process in the target country, native in the target language, fluent in the source language	Year of translation	Translator from professional agency	Translator health care professional[a]	Number of issues solved to establish semantical equivalence with the source language	
						Child-report	Parent-report
**United Kingdom English[a]**	Swedish	Recruited translator with health care profession who was not part of the research team but from 2019, it was Natalie Durkin. From 2019, Corné de Vos carried on with the South African English version, using the UK version as basis.	2015-2017	Yes	Yes	10	10

**US English**	Swedish	Recruited translator with health care profession who was not part of the research team. From 2018, Benjamin Zendejas Mummert	2015-2017	Yes	Yes	10	10
**Turkish**	Swedish	Tutku Soyer	2018	No	Yes	4	6
**European Spanish**	English	Alba Sánchez Galán	2019	Yes	No	2	8
**Hungarian**	English	Katalin Eszter Müller	2019	No	Yes	10	9
**Polish**	Swedish	Anna Rozensztrauch	2020	Yes	Yes	6	7
**Croatian**	Swedish	Ivana Sabolic	2020	Yes	No	8	9
**Mandarin Chinese**	English	Siqi Li	2020	No	Yes	5	5
**Norwegian**	Swedish	Kjersti Birketvedt	2021	No	Yes	8	6
**French**	Swedish	Anastasia Fourtaka	2021	Yes	No	8	8
**Mexican-Spanish**	Swedish	Juan Domingo Porras-Hernandez	2022	Yes	Yes	10	10

[a]used in United Kingdom and South Africa

Reference as cited in the main manuscript

45. Li, S., Dellenmark-Blom, M., Zhao, Y., Gu, Y., Li, S., Yang, S., Quitmann, J. H., & Huang, J. (2022). The Chinese Mandarin Version of the Esophageal-Atresia-Quality-of-Life Questionnaires for Children and Adolescents: Evaluation of Linguistic and Content Validity. Int J Environ Res Public Health, 19(22).
